# Evaluation of an untargeted nano-liquid chromatography-mass spectrometry approach to expand coverage of low molecular weight dissolved organic matter in Arctic soil

**DOI:** 10.1038/s41598-019-42118-9

**Published:** 2019-04-09

**Authors:** Mallory P. Ladd, Richard J. Giannone, Paul E. Abraham, Stan D. Wullschleger, Robert L. Hettich

**Affiliations:** 1 Bredesen Center for Interdisciplinary Research & Graduate Education, University of Tennessee, Knoxville, TN 37996 USA; 2 Chemical Sciences Division, Oak Ridge National Laboratory, Oak Ridge, TN 37830 USA; 3 Environmental Sciences Division, Oak Ridge National Laboratory, Oak Ridge, TN 37830 USA

## Abstract

Characterizing low molecular weight (LMW) dissolved organic matter (DOM) in soils and evaluating the availability of this labile pool is critical to understanding the underlying mechanisms that control carbon storage or release across terrestrial systems. However, due to wide-ranging physicochemical diversity, characterizing this complex mixture of small molecules and how it varies across space remains an analytical challenge. Here, we evaluate an untargeted approach to detect qualitative and relative-quantitative variations in LMW DOM with depth using water extracts from a soil core from the Alaskan Arctic, a unique system that contains nearly half the Earth’s terrestrial carbon and is rapidly warming due to climate change. We combined reversed-phase and hydrophilic interaction liquid chromatography, and nano-electrospray ionization coupled with high-resolution tandem mass spectrometry in positive- and negative-ionization mode. The optimized conditions were sensitive, robust, highly complementary, and enabled detection and putative annotations of a wide range of compounds (*e*.*g*. amino acids, plant/microbial metabolites, sugars, lipids, peptides). Furthermore, multivariate statistical analyses revealed subtle but consistent and significant variations with depth. Thus, this platform is useful not only for characterizing LMW DOM, but also for quantifying relative variations in LMW DOM availability across space, revealing hotspots of biogeochemical activity for further evaluation.

## Introduction

Low molecular weight (LMW, 50–1500 Da) dissolved organic matter (DOM) is the most accessible fraction of soil organic matter to microbial decomposers and thus, most susceptible to mineralization and release as greenhouse gases such as carbon dioxide (CO_2_) and methane (CH_4_)^1^. In terrestrial systems undergoing rapid change due to warming temperatures, such as the Arctic, LMW DOM (*i*.*e*. primary metabolites, amino acids, sugars, lipids, peptides) represents a detailed chemical snapshot of organic matter vulnerability that could help improve predictions of where (hotspots) this carbon release is more likely to occur^2–4^. However, the heterogeneity of this pool coupled with consistently low concentrations due to high turnover rates pose significant challenges in detection and quantitation. As such, most analyses of LMW DOM in Arctic soil have been at the bulk level (i.e. total organic carbon, separation by physical or chemical fractionation)^5,6^ or have targeted a specific subset of compounds—largely, amino acids^7–9^.

Several studies have demonstrated how utilizing an untargeted approach reveals a broad range of DOM compounds by nuclear magnetic resonance spectroscopy^10^, ultraviolet-visible or excitation-emission matrix fluorescence spectroscopy^11^, or gas chromatography/mass spectrometry measurements^12,13^. Due to inherent limitations associated with these techniques however, including detection sensitivity, dynamic range, difficulties with very complex mixtures, or a need for chemical derivatization prior to analysis, there has been increased interest in evaluating high-resolution mass spectrometry (HRMS) approaches^14,15^ for the characterization of LMW DOM in soil—in particular, liquid chromatography coupled with electrospray ionization (LC-ESI-MS)^16,17^.

In recent years, LC-ESI-MS has become a powerful analytical tool for obtaining broad coverage of chemically-complex mixtures of small molecules, including environmental samples^18–20^, due its sensitivity, wide detection range, and high throughput capabilities, which makes it an attractive alternative for the characterization of DOM in soil. While reversed-phase (RP) liquid chromatography in positive MS-ionization mode has dominated untargeted metabolomics studies, the limitations of using a single chromatographic phase or polarity have also been documented^21^; especially when analyzing mixtures with a high fraction of water-soluble, highly-polar metabolites^22,23^ like DOM, as these compounds are not well-retained by RP^24^. Hydrophilic interaction liquid chromatography (HILIC) however, has been shown to be an effective tool for retaining and separating small, highly-polar compounds, thereby enabling relative quantitation^25–27^. In addition to combining multiple LC techniques, adding negative-mode ionization has also been shown to expand metabolome coverage in bacterial cultures, plant and human tissue, and urine^28–31^. Although applied extensively in other systems, the use of untargeted LC-MS platforms to characterize DOM from soil is still in its infancy^16,17,32–34^, and to the best of our knowledge, no dual-LC, dual-polarity untargeted metabolomics approach has yet been examined for characterization of the range of LMW DOM from Arctic soil.

Arctic soils present several unique analytical measurement challenges. For one, they are generally water-logged due to the presence of permafrost, or permanently frozen ground, beneath the active layer (layer of soil that thaws seasonally), which mobilizes DOM and can lead to low concentrations due to it being transported (either vertically or horizontally) out of the system. In addition, these soils often experience frequent freeze-thaw events, creating both aerobic and anaerobic environments in close proximity (down to the microaggregate scale), which leads to diverse microbial communities^35^ and substrate pools, and high turnover rates^36^. Finally, low temperatures in the Arctic have slowed decomposition, which has led to high organic content as well as heterogeneous DOM chemistry, including spatial variations in pH, redox status, and/or age which impacts biogeochemical cycling and decomposition rates^37–40^.

Here, we evaluate RP- and HILIC-ESI-MS in positive- and negative-ion modes for the characterization of LMW DOM from Arctic soil water extracts, and then apply the optimized technique along the length of an Arctic organic horizon to examine the capabilities of the approach in determining relative abundance differences across space. Using a data-dependent approach, high-resolution tandem mass spectrometry (HRMS/MS) experiments were carried out, adding a third dimension (RT, MS^1^, and MS^2^) for annotation^41^ and flexibility in the technique to examine both known (already listed in a database) and unknown compound structures^42^. Finally, because soils can have high salt concentrations which results in substantial ion suppression at the macro-scale^16^, we employed a nano-scale LC column/emitter and flow rates to enhance sensitivity and enable more accurate relative quantitation^43,44^. Establishing this methodology and benchmarking its performance in Arctic soils for the first time lays the technical foundation for future studies aiming to incorporate LMW DOM molecular data into process-based ecological models^45,46^.

## Results and Discussion

**Figure 1:**
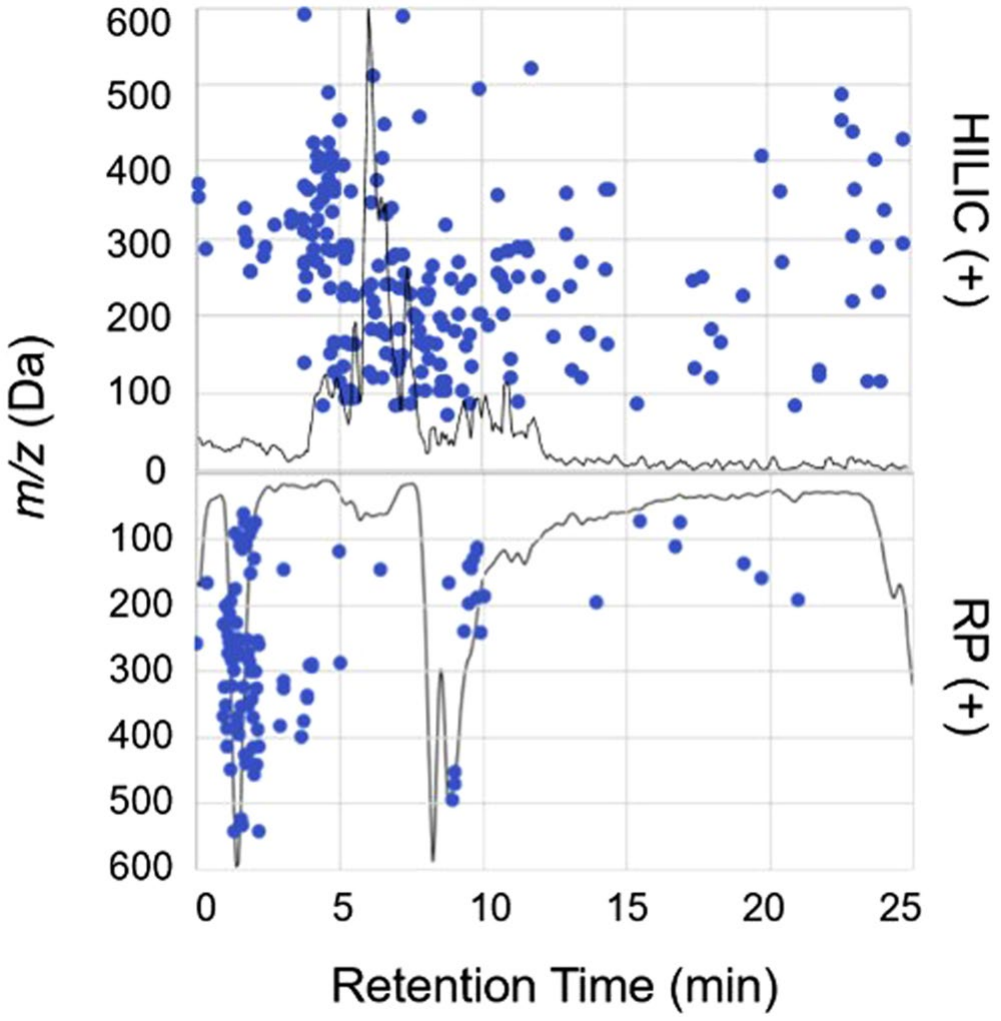
Scatter plot of the features detected (intensity >1.0E4, +/−0.005 *m/z*) in a single soil water extract and the elution profiles for HILIC (top) and RP (mirrored bottom) in positive-ion mode demonstrating different separation profiles of LMW DOM on each LC phase. Each marker matches to a *m/z* and retention time (RT). The corresponding normalized base peak chromatograms are overlaid on top to show a typical elution profile for each LC condition and display trends between *m/z* and RT. Additional examples in negative-ion mode are shown in Fig. S4.

**Figure 2:**
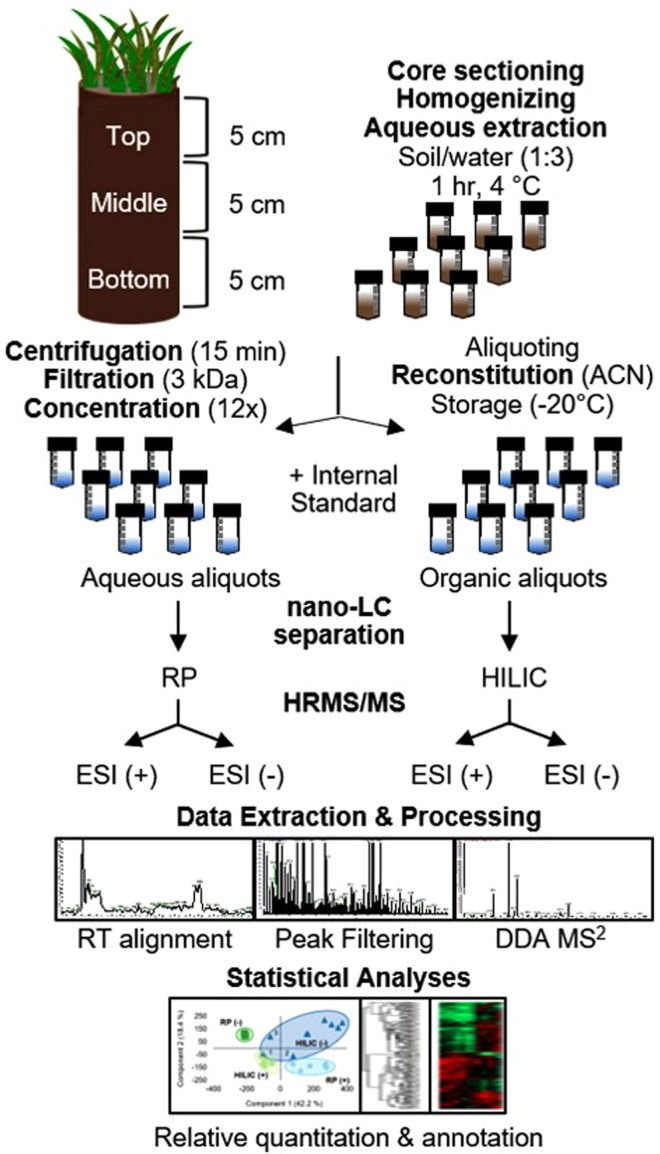
Schematic of the untargeted metabolomics approach established and applied in the present study for the analysis of LMW DOM from Arctic soil water extracts. After the filtration step, triplicate extracts for each section of the core (n = 9) were split and handled separately. The resulting concentrated aliquots (18 samples) were run on two LC phases and in two MS polarities, resulting in four analytical conditions per sample. RT: retention time. ESI (+/−): electrospray ionization positive or negative mode. DDA: data-dependent acquisition (unbiased precursor selection for MS^2^ fragmentation).

The goal of this work was to optimize and demonstrate a sensitive, high-throughput, untargeted approach to detect, quantify (relative), and putatively annotate variations in LMW DOM availability across space in Arctic soil. A preliminary analysis of Arctic soil water by RP-MS revealed that although some compounds were retained effectively, eluting later in the run, a majority (~80%) of the most abundant ions (intensity >5.0E4) were observed with minimal retention (RT < 2 min), and a maximum molecular weight of ~600 Da (Fig. 1). This is consistent with the emerging view that much of dissolved soil organic matter is comprised of plant- or microbial-derived LMW compounds^47^ that are often polar and therefore not well-retained by RP. To mimic native soil-water LMW DOM chemistry and obtain a sample most consistent with compounds that are available to microbial communities^33,48^, we examined a single, aqueous extraction. Then, to enable characterization and expand coverage, we evaluated four nano-LC-MS analysis conditions—HILIC (+), HILIC (−), RP (+), and RP (−). Each step of the final workflow (Fig. 2) was optimized to maximize throughput, enhance the signal strength of low abundant analytes, and minimize introduction of non-analyte signals which complicate annotation. The optimized approach was evaluated based on its reproducibility, separation power, and both the qualitative and quantitative performance when applied to triplicate extracts from three assigned depths—top (samples 1–3), middle (samples 4–6), and bottom (samples 7–9)—along a single organic horizon obtained from an Alaskan Arctic landscape. While additional cores and standards would be necessary for an ecological study, absolute quantitation, or identification of compounds, these fall outside the scope of the study designed here, which was to evaluate the optimized untargeted metabolomics approach to explore the range of LMW DOM in a new and complex matrix.

### Optimization of nano-HILIC-MS analysis

Given that most LC-MS-based metabolomics analyses have used RP, were carried out at the macro-scale, or have been applied in alternate sample matrices^20^, optimizing and evaluating the nano-HILIC conditions for the separation of LMW compounds from soil water was first required. Here, we chose to exploit a zwitterionic, polymer-based HILIC material (ZIC-pHILIC) that has demonstrated improved reproducibility over other HILIC phases, and a higher tolerance for both acidic and alkaline conditions (pH range 2–10), enabling a multiple ionization strategy to be employed^49^. Optimization was carried out using a mixed standard of fifteen LMW organic compounds of varying sizes and chemical properties (Table S1).

#### Sensitivity and mass accuracy

To evaluate the retention of various LMW DOM compounds on the HILIC column, their electrospray ionization efficiencies, and probe detection limits and interferences, a mixed standard curve (equimolar, 10 ng mL^−1^ – 10 µg ML^−1^) was run neat (directly on the column without any other matrix), and also spiked into and then extracted from Arctic soil at ecologically-relevant concentrations^50^ and analyzed by nano-HILIC-MS. All compounds were detectable (S/N > 3) and reliably quantified at 10 ng mL^−1^ or better when extracted from the soil matrix, except for N-acetyl glucosamine and urea, which were detectable at 100 ng mL^−1^ (Fig. S1). This suggests that sugars may not have ionized as well under the conditions employed here and further optimization would be necessary to detect the broad range of sugars that are commonly found in soils. In addition to each of the compounds demonstrating varying ionization efficiencies (Fig. S1a), the optimized approach was able to detect variations in extraction efficiency as well, as demonstrated by the broader spread of peak heights and shallower gains in signal with increasing concentration when extracted from soil (Fig. S1b). While extraction efficiency would need to be determined for each compound for absolute quantitation, the aim of the study here is to evaluate variations in the relative availability across space in soil. In addition, despite the variations in ionization and extraction efficiency among compounds, in both scenarios, the signal response curves exhibited a linear gain in signal over at least two orders of magnitude with average Pearson correlation coefficient (R^2^) of 0.9946 and 0.9924 for the neat and spiked standards, respectively, demonstrating a broad dynamic range for the detection of LMW DOM analytes by this technique. Each standard was detected <10 ppm mass error (Table S1), demonstrating the mass accuracy of the MS technique and reliability of the measurement for post-acquisition peak clustering and putative annotation.

#### Chromatographic reproducibility

To evaluate the performance of the columns, an internal standard (10 µg mL^−1^) was added to triplicate extracts (see Materials and Methods for more detail) from each of the three soil core depths (n = 9). While it has been reported that HILIC columns often suffer from more variable peak shapes and shifting retention times^51^, the RT deviation observed here, across all nine extracts, was <1.8 min (CV = 12.7%) (Fig. S2), comparable to a recent study that used the same ZIC-pHILIC material for soil extracts^17^ and better than the C18-RP column employed here. In addition, this variation was within the bounds of what can be corrected by the RT alignment algorithm in the data processing software (see Materials and Methods). Peak areas for the internal standards also showed reasonable quantitative reproducibility among replicates (CV_avg_ < 15%) for each LC-MS condition (Fig. S3), consistent with recent studies that have also used LC-MS for untargeted metabolomic profiling in complex biological matrices^22,30^.

It should be noted that the HILIC column needed more time for pre-conditioning and re-equilibration to achieve a stable background, and some peak tailing was observed (Fig. S2). This is likely due to competition between the primary aqueous-partitioning retention mechanism and secondary electrostatic interactions with the zwitterionic sulfobetaine group on the surface of the ZIC-pHILIC stationary phase. Nevertheless, the HILIC column demonstrated markedly improved separation and peak shape for LMW DOM analytes when compared to the RP column, highlighted by the greater distribution of features eluting over the full gradient and sharper peak shapes in both positive- and negative-ion modes (Figs 1 and S4).

### Evaluation of untargeted method performance for LMW DOM in Arctic soil

#### LMW DOM coverage

**Figure 3:**
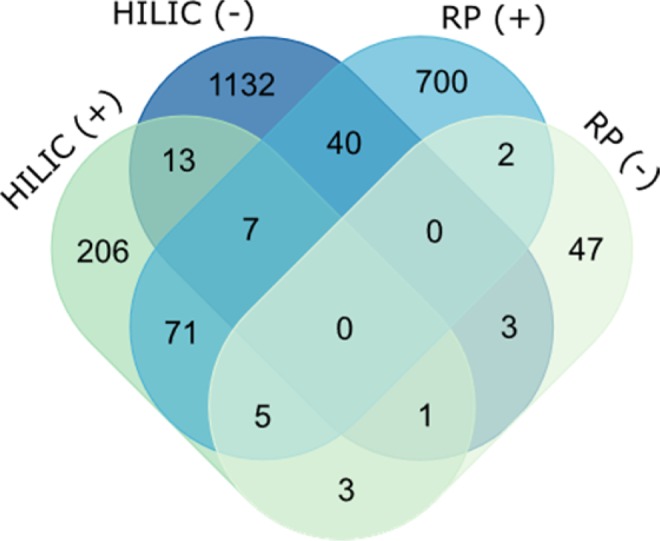
Overlap of HQFs detected by HILIC and RP in positive- and negative-ion MS polarities (based on the MS^1^ neutral mass for the corresponding [M + H]^+^ or [M − H]^−^ ion, +/−0.001 Da).

Expanding the number of analytes detected is central to any metabolomics study and to obtaining as unbiased and comprehensive of a measurement as possible. Across the 36 analytical runs (9 extracts, 4 LC-MS conditions) plus the blank and control runs, 12,924 features were detected (Table 1). After removing features that were observed in the blank or control (intensity >1.0E5) and features that resulted in zero peak area after normalization (see Materials and Methods for more detail), the total number of high-quality features (HQFs) was 3,690. HILIC (−) produced the most HQFs with 1,705, accounting for 46% of all HQFs observed, followed by RP (+) with 1,462 (40%), HILIC (+) with 438 (12%), and finally RP (−) which detected 85 (2%) (Table 1). The paucity of LMW DOM analytes detected by RP (−) is likely due to poorer retention and less favorable ionization conditions. By taking each singly-charged precursor ion (+/−0.001 *m/z*) to its neutral mass and analyzing the overlap between conditions (Fig. 3), it was observed that HILIC (−) and RP (+) detected the most HQFs with 1,132 and 700, respectively. While these two conditions accounted for 88% of the dataset, the four optimized techniques were still highly orthogonal with just 4% (145 features) detected by more than one condition at this high-resolution threshold (+/−0.001 Da), illustrating the benefits of combining RP and HILIC, and positive- and negative-ion modes to expand coverage of the LWM DOM pool.

**Table 1:** LMW DOM coverage by HILIC and RP in positive- and negative-ionization modes at each level of data filtering, expressed as the number of features detected across all nine soil water extracts.

LC-MS Condition	All Features	High-Quality Features	Unique HQFs^a^	Abundant HQFs^b^	Varied significantly with depth^c^	Significant HQFs with MS^1^ match (+/−5 ppm)
HILIC (+)	1455	438	206	247	164	35
HILIC (−)	8343	1705	1132	257	79	14
RP (+)	1828	1462	700	202	12	8
RP (−)	1298	85	47	10	2	2

^a^Unique high-quality features observed by only one LC-MS condition, determined by examining the overlap of the neutral precursor masses (+/−0.001 Da). ^b^Abundant features were observed in at least 2 of 3 extraction replicates at each depth above an intensity threshold of 1.0E5 ion counts. ^c^Abundant features with differential abundances that varied significantly (log_2_ fold change >1.5, p-value < 0.05) between soil core depths.

#### Measurement depth

**Figure 4:**
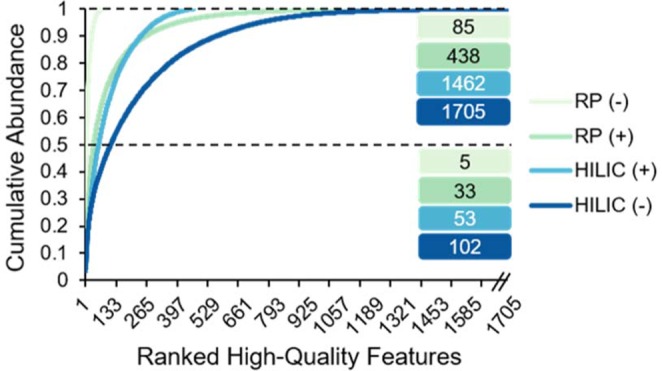
High-quality features ranked by abundance (1 = most abundant, 1705 = least abundant) and the relative contribution of each to the cumulative abundance demonstrating the depth of measurement for each LC-MS condition evaluated. The number of LMW DOM features detected by each LC-MS condition accounting for half and the total cumulative abundance are reported.

In addition to expanding the number of compounds detected, an untargeted technique should be able to reliably detect both high- and low-abundant signals. This is especially true for Arctic soils, where low-abundant DOM signals could indicate a greater biological importance, in that lower concentrations may suggest a microbial preference for those substrates and that they are cycled through the soil at a faster rate, thereby contributing disproportionately to the fraction of SOM that is mineralized into CO_2_ and CH_4_^52,53^. To explore the sensitivity and dynamic range of the untargeted approach developed here, we examined the proportion for which each HQF contributed to the total signal of HQFs detected by each LC-MS condition. HILIC detected more low-abundant features than RP, consistent with it having more favorable retention and ionization conditions leading to enhanced MS detection sensitivity^54^. For example, while only 5 features made up 50% of the signal for RP (−), 102 different features accounted for the same proportion on the HILIC column (Fig. 4).

#### Analytical reproducibility

Using a unique identifier and corresponding normalized peak area for each HQF, we evaluated the reproducibility of the untargeted measurement across extraction replicates using principal component analyses (PCA). When comparing the nine samples and three controls for each LC-MS condition, a strong separation was observed (Fig. S5) providing additional evidence that the variation observed in the LMW DOM profiles was nonsystematic, but instead related to biogeochemical variation with depth. One outlier (sample 5) separated apart from both the other samples and the controls, but after careful evaluation of the soil sample, the experimental conditions, and the resulting data, it was not immediately obvious what was driving that separation, and thus further analysis would be warranted for a more detailed study. PCA also revealed separation between the four LC-MS conditions (Fig. S6) further demonstrating their orthogonality. HILIC (−), which detected the highest number of HQFs, showed the most variation across the nine extractions, while RP (−), which detected the fewest, showed the least amount of variation. Interestingly, the three extraction replicates within the HILIC (−) dataset that stood out from the other six, clustered closer to the other three LC-MS conditions and corresponded to samples 1–3 from the top section of the horizon. These data suggest that at the top of this organic horizon, there may exist a common set of abundant, amphiphilic compounds that ionize in both MS polarities that do not get transported deeper into the soil profile.

Overall, the number of features detected by the combined LC-MS conditions and the reproducibility of the untargeted measurements across extraction replicates demonstrates the robustness of the workflow developed here. Substantially more information (60% more features) was obtained by integrating HILIC and negative-ionization mode, emphasizing the complementarity of the optimized LC-MS conditions and the ability of this untargeted technique to expand coverage of LMW DOM in these complex, organic-rich soils.

### Application of the untargeted metabolomics approach to evaluate relative variations in LMW DOM availability with depth

**Figure 5:**
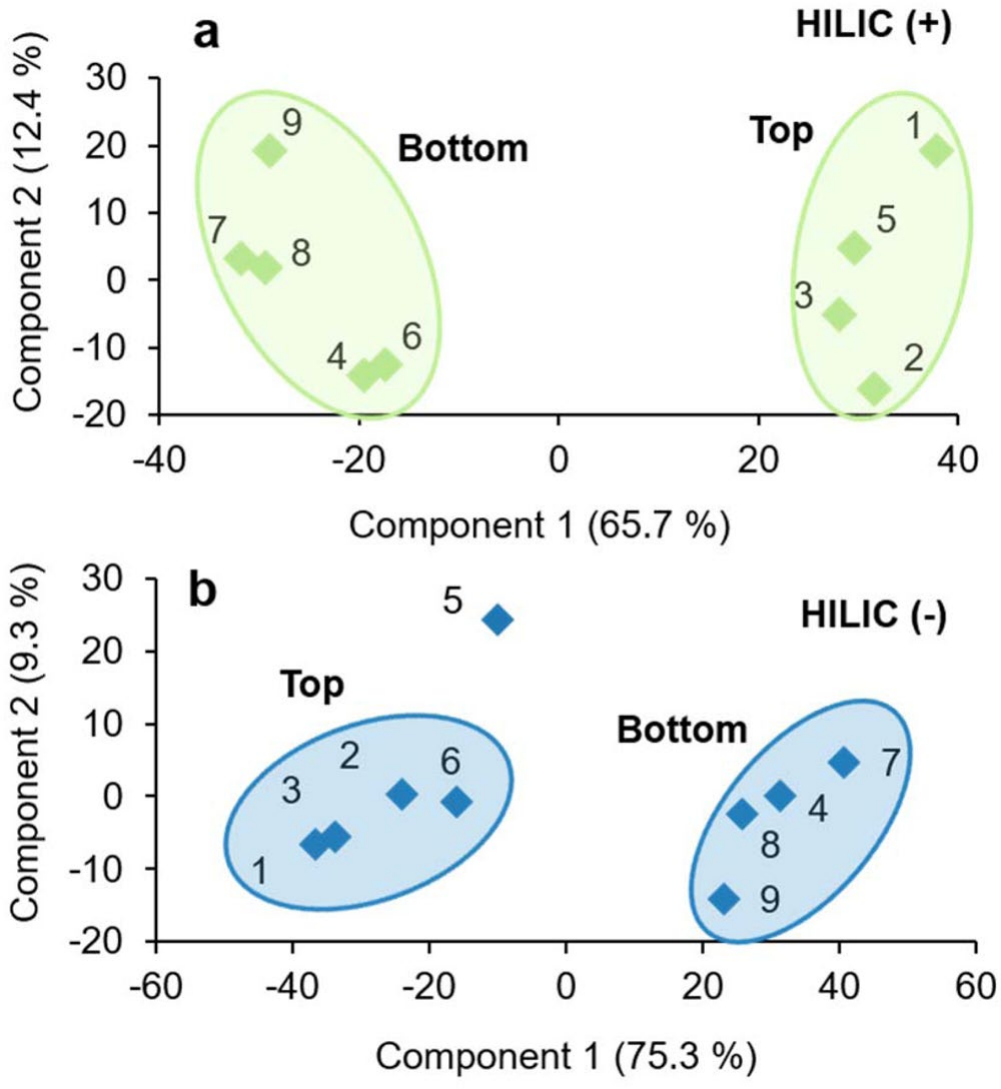
Principal component analyses of high-quality features detected in soil water extracts analyzed by (**a**) HILIC (+) and (**b**) HILIC (−) demonstrating the sensitivity of the untargeted technique to detect subtle variations in LMW DOM across space in these organic-rich soils.

After filtering the data to identify the most reproducible, “abundant HQFs” (see Materials and Methods), HILIC was found to have detected a total of 247 and 257 features in positive- and negative-ion modes, respectively, while RP detected 202 in positive-ion mode and 10 in negative-ion mode (Table 1). RP (−) had less favorable mobile phase conditions and more variable chromatography which likely led to weaker ionization, lower intensities, and fewer reproducible features. By examining the PCA for each condition separately, we found that even though the soil core represented a single horizon (organic) and would be represented as such in most biogeochemical models, the untargeted approach evaluated here revealed a finer-level spatial heterogeneity in LMW DOM availability along the length of the horizon (Fig. 5). However, instead of separating into three distinct groupings as one might expect based on our operationally-defined depths, only two groups emerged, suggesting this seemingly-homogenous organic horizon would more accurately be described as having two distinct layers due to biogeochemical variation, indicated by measurable differences in the LMW DOM profiles.

#### Relative quantitation and putative annotation of LMW DOM features that varied across space

**Figure 6:**
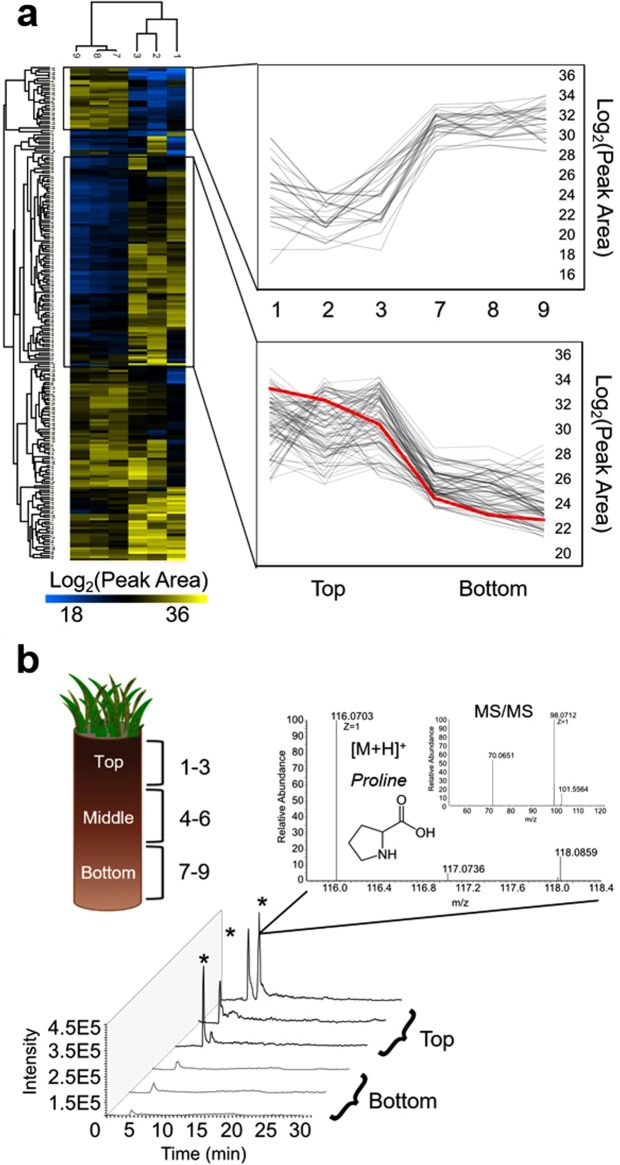
(**a**) Heatmap of the unique IDs and normalized log_2_ peak areas for each abundant HQF detected by HILIC (+). The dendrogram to the left of the heatmap clusters LMW DOM metabolites that varied similarly between the top and bottom of the soil core. To the right, we call out two clusters that starkly increased or decreased with depth after normalizing to per gram dry weight soil. One feature that decreased with depth, highlighted in red, is further analyzed in (**b**). (**b**) Cross-sectional diagram of the soil core showing three depth increments and corresponding extraction replicate sample numbers. Stacked extracted ion chromatograms and mass spectra (MS^1^ and MS^2^, insets) for a feature (116.0703 *m/z*) detected by HILIC (+) at RT ~6.1 min. The feature was detected (intensity >1.0E5) in all six soil extracts but not in the blanks or controls. Extraction triplicates yielded similar amounts (CV < 3%) despite some peak splitting, and there was a 4-fold difference between the log_2_ peak areas of the top and bottom sections of the core (p-value < 0.05 by t-test). The feature was putatively identified as proline by matching the MS^1^ spectrum in MMCD and confirming with the MS^2^ spectrum in MassBank.

To visualize more detailed patterns of LMW DOM availability along the length of the horizon, two-way hierarchical clustering using heatmaps was performed on the abundant HQFs detected by each LC-MS condition. An example of this is shown in Fig. 6a using the HILIC (+) dataset. The extracts from either depth (top or bottom) clustered together, indicated by the top dendrogram, and metabolites that varied similarly with depth were clustered together, shown in the dendrogram to the left. While there were LMW DOM features that were equally abundant across the entire length of the horizon, differences in the normalized peak areas were especially apparent for two clusters that either increased or decreased from the top to the bottom of the horizon (Fig. 6a, insets). While replicate cores and additional data (*i*.*e*. compound-specific extraction efficiencies, biomass and DOC content) would be valuable, our results alone already demonstrate the ability of the optimized approach to detect subtle variations in the availability of LMW DOM between replicates and across space in soil. In addition, to generate a more ecologically-relevant list of features for annotation, we normalized peak areas to per gram dry soil and identified which abundant HQFs varied significantly (log_2_ fold change >1.5, p-value < 0.05 by t-test) between the top and bottom of the horizon, indicating a change in availability due to biological variation. The number of features that met these criteria for each LC-MS condition are reported in Table 1. HILIC (+) and (−) detected the highest number of differentially-abundant LMW DOM features with 164 and 79, respectively, while the RP conditions detected 14 in total, demonstrating that the conservative thresholds applied here helped ensure a robust measurement.

The features that varied consistently and significantly with depth were searched against multiple freely-available online databases using high-mass accuracy (<5 ppm) MS^1^ measurements. When compounds matched to multiple database hits, possible matches were examined in an iterative approach by comparing the experimental fragmentation pattern (MS^2^) with available data (see Supplementary Information). It is important to note that because the MS^2^ spectra in the various databases were often collected at different CID energies than the experimentally-obtained MS^2^ spectra here, there were varying degrees of similarity among the putatively annotated compounds, and thus confirmation with an authentic standard at the same CID energy on the same system would be necessary to make a full identification. While there are numerous examples of fragmentation interrogation in the supplementary figures, one example of this has been highlighted in Fig. 6.

The feature (highlighted in red in Fig. 6a) eluted in the void volume on the RP column but was effectively retained (RT 6.1 min) by the HILIC column, further supporting the use of dual-chromatographic separations for the analysis of LMW DOM from soil (Fig. 6b). The feature was detected in positive-ion mode ([M + H]^+^ = 116.0703 *m/z*) reproducibly across replicates (CV < 3.0%) and decreased significantly (4-fold log_2_ change, p-value < 0.05) with depth. The MS^1^ accurate mass matched to multiple hits in the MMCD and HMDB databases but was putatively annotated as proline by comparing the MS^2^ spectrum to available data in MassBank (Fig. 6b), emphasizing the value of collecting data-dependent MS^2^ fragmentation data. Proline is an amino acid and osmolyte that accumulates in microorganisms and plants to help protect against stresses such as the drying and rewetting of soils^55,56^. That it was detected appreciably in the LMW DOM pool in soils that were collected from a saturated, low topographical area (*i*.*e*. not drought stressed) on the landscape may suggest that it had accumulated due to an increase in protease activity coupled with reduced uptake by plants/microbes^57^, or enhanced exudation of excess proline from plant roots (*i*.*e*. priming) or microbial turnover, possibly due to alkaloid/salt stress^58,59^. The decrease in this LMW DOM metabolite with depth may indicate that it is taken up by plants and/or microbes deeper in the soil profile, consistent with recent findings that the direct uptake of organic nitrogen may become more important in nitrogen-limited environments like Alaskan tundra systems^60,61^. This example demonstrates the capabilities of this untargeted, hypothesis-generating approach at identifying hotspots of biogeochemical variation for further analysis. A full list of the LMW DOM compounds that were putatively annotated in this way, using high-resolution MS^1^ and MS^2^ data within an average mass error of 3.3 ppm, can be found in the supplementary information (Table S2).

Of the HQFs that consistently and significantly varied between depths, 59 (23%) were putatively annotated and 198 (77%) were unmatched, highlighting a critical advantage of our approach—the ability to detect previously uncharacterized compounds that vary across space due to some biogeochemical process, thus providing targets for further inquiry. For example, one unmatched feature was retained by HILIC (−), detected reproducibly across replicates (CV < 5%) at RT 22.7 min with an accurate mass of 281.1440 *m/z,* and was found to increase significantly (7-fold, p-value < 0.0007) with depth. Analyzing the high-mass accuracy fragmentation data, neutral losses of 43.9897 *m/z*, 18.0106 *m/z*, and 14.0155 *m/z* were observed; likely a carboxylic acid group, water loss, and methylene group respectively, emphasizing the utility of this technique to provide structural information about unknown LMW DOM compounds. Molecular networking for untargeted -omics datasets is a growing area of research in the mass spectrometry community^62–64^, and leveraging high-resolution MS^2^ fragmentation information like this can assist in grouping unknown compounds based on their structural (spectral) similarity.

The 59 compounds putatively annotated from a single water extract ranged in polarity and aromaticity, from plant and microbial metabolites to organic acids, osmolytes, sugars, lipids, and simple peptides (Table S2), yielding insights into the chemical diversity and reactivity of LMW DOM in Arctic soil water detected by the optimized platform. As with any untargeted approach, the number of features annotated depends on the data analysis thresholds and the level of curation of each database. As such, the features listed here do not represent all LMW DOM molecules that can be detected by the described technique. It’s important to note that our aim was not to identify each feature detected but instead to benchmark the analytical performance of the untargeted approach in this unique and complex matrix, demonstrate the value of the approach in revealing an information-rich molecular profile of LMW DOM availability in soil, and to analyze how this approach may be used to evaluate variations in those profiles across space (here, with depth). Further examination of feature clusters that varied similarly and significantly with depth would likely reveal additional biogeochemical processes impacting the availability of these compounds. In addition, follow-up targeted analyses (*e*.*g*. isotopic or flux analyses) could be carried out for absolute quantitation of LMW DOM analytes-of-interest or to monitor a specific metabolic pathway (e.g. methanogenesis) over time for example.

Ultimately, these results demonstrate an optimized untargeted metabolomics approach for the analysis of LMW DOM from Arctic soil water extracts. The approach evaluated here was high-throughput, sensitive, and robust, with a high tolerance for salts, and could feasibly be applied in a broad range of soils. The nano-LC-MS conditions were highly complementary and revealed a broad diversity of small molecules in Arctic soil water extracts. LMW DOM profiles were reproducible and distinguishable between samples, allowing for the spatial variability of these organic substrates to be observed at the molecular level. Even subtle differences in the relative abundance of features with depth were detected with robust data mining strategies, highlighting the potential of the LMW DOM pool to be used to identify biogeochemical hotspots in soils. Reproducible retention profiles and high-mass accuracy molecular and fragmentation data provided both qualitative and relative quantitative data, yielding an information-rich chemical snapshot of biogeochemical activity in soil. Future work should include applying the technique across multiple cores or in different systems, correlating shifts in LMW DOM chemistry with microbial community composition and environmental variables to assist with mapping LMW DOM compounds to metabolic pathways. Furthermore, this approach could be leveraged in larger-scale studies to provide insight into LMW DOM origins and transformations over space or time and detailed stoichiometric information for mechanistic models, helping to reduce uncertainty in predictions of how Arctic landscapes will respond under a warmer climate.

## Materials and Methods

A schematic of the experimental workflow established in this study is shown in Fig. 2.

### Chemicals

A description of mobile phases, solvents, and a list of authentic standards used to evaluate the optimized approach developed here can be found in the Supplementary Information.

### Sample collection and soil processing

A soil core horizon (10 cm diameter, ~15 cm depth) was collected from the organic-rich active layer of a saturated, continuous-permafrost landscape on the Barrow Environmental Observatory, AK (71° N, 156° W) and shipped frozen to Oak Ridge National Laboratory (Oak Ridge, TN) where it was stored at −80 °C until processing. The frozen core, representing a single organic horizon, was cut into three, 5 cm sections using a band saw. Each section—defined here as top, middle, or bottom—was thawed at 4 °C overnight and then homogenized by hand, removing any mineral, inorganic, or live plant material^65^.

### Optimized LMW DOM extraction

Soils were extracted in triplicate with LC-MS-grade H_2_O (pH = 5.0, 1:3 w/v) in 50 mL centrifuge tubes (VWR) at 4 °C on a standard orbital shaker (VWR, Model 1000) at ~120 rpm for 1 h, resulting in three extracts per depth (9 total) to be analyzed by nanoLC-MS. Three controls were prepared by adding LC-MS-grade H_2_O to centrifuge tubes with no soil to undergo the same extraction procedure. Extracted soils and controls were centrifuged (Eppendorf Centrifuge 5804 R) at 4 °C and 4500 rpm for 15 min and the supernatant was then transferred to pre-rinsed centrifugal filter units (Amicon Ultra, 3 kDa) for concentration. The filtered extracts were evaporated down to 0.5 mL (12x concentration) in a Thermo Savant SC210A SpeedVac Concentrator and separated into two 0.25 mL aliquots. One aliquot was further evaporated to near-dryness and brought back up to 0.25 mL in 95:5 (v/v) acetonitrile:water, creating one organic and one aqueous aliquot per sample for analysis by HILIC and RP, respectively. Although no heat was applied, any volatile compounds that came out of solution during vacuum evaporation would not be included in this analysis.

### Nano-LC-MS/MS analyses

Measurements of standards (Table S1) and samples were carried out using a Dionex UltiMate 3000 HPLC pump (ThermoScientific) coupled to an LTQ-Orbitrap Velos Pro mass spectrometer (ThermoFisher) equipped with a nano-electrospray ionization source (Proxeon, Denmark) operated in positive- or negative-ion mode under direct control of the XCalibur software, v2.2 SP1.48 (ThermoFisher). Detailed source conditions and instrument parameters can be found in the Supplementary Information.

Extracts were thawed and prepared immediately prior to injection by adding either 0.1% formic acid (FA) or ammonium hydroxide (NH_4_OH) to help with ionization, and either 6-methylaminopurine riboside (6-MAP) or adenosine (final concentration, 10 µmol L^−1^) as an internal standard for positive- or negative-ion mode, respectively. Internal standards were added to evaluate method performance and reproducibility, and to assist with retention time (RT) alignment and annotation of LMW DOM^66^. Analyses were randomized to minimize instrument-derived variation, and technical blanks representing the column re-equilibration conditions were run regularly to monitor background ions and carry-over.

Separations were performed on 100 µm i.d. fused-silica (Polymicro Technologies) columns, which were laser-pulled in-house and pressure-packed to 20 cm with either Kinetex C18 resin (5 µm, 100 Å, Phenomenex) or zwitterionic, polymer-based ZIC-pHILIC resin (5 µm, Sequant, bulk material kindly provided by EMD Millipore) resulting in four separate LC-MS analyses per sample. Mobile phase compositions, gradient conditions, and MS parameters were systematically adjusted to provide the best ESI spray stability, signal strength, LC peak shape, and separation. Only mobile phase additives that were compatible with the ESI source were examined (Table S3). Thus, ion-pairing agents and non-volatile buffers were excluded from method development. The final gradients used for each LC-MS condition are listed in Table S4. Prior to MS analysis, each column was washed off-line for 1 h with an alternating gradient from 100% A to 100% B, but not exceeding 60% aqueous solvent on the HILIC columns so as not to disrupt the aqueous layer on the surface of the stationary phase^25,67^. Each column was positioned on the nano-spray source, aligned in front of the MS capillary inlet. Samples or standards were manually injected directly onto the column using a 1 µL fused-silica loop, and nano-flow rates were achieved with a split-flow setup prior to the injection loop. The pump was set to 0.1 mL min^−1^, measuring ~250 nL min^−1^ at the tip. A post-gradient wash was applied at the end of each run to ensure column re-equilibration, and to maintain the ionic strength of the HILIC material.

### Data Extraction and Processing

Raw LC-MS data were subjected to peak picking, alignment, and normalization using the open-source MZmine2 (v2.28) software^68^. The optimized module parameters and data filtering strategy are described in detail in the Supplementary Information. Briefly, the resulting matrix of features—defined here as a unique RT, MS^1^ *m/z*, and MS^2^ fragmentation spectrum—was filtered to remove any artifact signals (features with intensity >1.0E5 in blanks or controls) that originated from sample collection, preparation, or analysis (i.e. extraction leachates, solvent contaminants, columns background)^69,70^. Integrated LC peak areas were obtained from the aligned extracted ion chromatograms and normalized to the internal standards using a ratio factor determined with the standard compound normalizer module in MZmine. While an internal standard for each feature detected is recommended for absolute quantitation, a standard specific to each ionization mode was applied here to evaluate the effectiveness of the technique at detecting relative quantitative variations across space and reduce the introduction of additional chemical species further complicating the chromatogram and/or mass spectrum^71,72^. Any features that had zero peak area after normalizing to the internal standard were also removed, resulting in a matrix of high-quality features (HQFs). The number and complexity of HQFs detected by each LC-MS condition were used to evaluate LMW DOM coverage, measurement depth, and the qualitative and quantitative reproducibility across replicates by comparing the accurate mass of the corresponding [M + H]^+^ or [M − H]^−^ molecular ion and the peak area for each feature. To evaluate the ability of the technique to detect variations in the availability of LMW DOM across space, raw peak areas were normalized to per gram dry soil and a more conservative list of only the most reproducible, “abundant HQFs”—observed in at least 2 of 3 extraction replicates at each depth above an intensity threshold of 1.0E5 ion counts—was generated. Finally, to generate a list of features for annotation, Student’s t-test and two-way hierarchical clustering (heatmaps) were used to examine relative abundance differences and identify LMW DOM analytes that varied significantly between depth increments.

#### Feature Annotation

Using a precursor mass tolerance of +/−5 ppm, significant features were putatively annotated using the open-source MZmine2 software and the MetaboSearch tool^73^, both of which search multiple, freely-available, online metabolite databases, including METLIN^74^, MMCD^75^, HMDB^76^, KEGG^77^, PubChem^78^, and LipidMaps^79^. Annotated compounds that matched to multiple MS^1^ hits in a database were manually scrutinized in an iterative approach by assessing high-resolution mass spectral data for consistent fragmentation profiles (see Supplementary Information). Only compounds that had a single MS^2^ match were included here.

## Supplementary information

Supplemental Information

## Data Availability

The high-resolution LC-MS data generated and analyzed in the current study can be found through the NGEE-Arctic data portal at 10.5440/1464956 ^80^.
