# Supplementary material for: Evaluation of an untargeted nano-liquid chromatography-mass spectrometry approach to expand coverage of low molecular weight dissolved organic matter in Arctic soil
Source: Sci Rep. 2019 Apr 9;9:5810. doi: 10.1038/s41598-019-42118-9 (PMC6456581; doi:10.1038/s41598-019-42118-9)
Supplement: Supplementary file 1 — Supplemental Information [file 41598_2019_42118_MOESM1_ESM.docx]

Evaluation of an untargeted nano-liquid chromatography-mass spectrometry approach to expand coverage of low molecular weight dissolved organic matter in Arctic soil

Mallory P. Ladd^1,2^, Richard J. Giannone^2^, Paul E. Abraham^2^, Stan D. Wullschleger^1,3^, Robert L. Hettich^1,2^*

^1^ Bredesen Center for Interdisciplinary Research & Graduate Education, University of Tennessee, Knoxville, TN, 37996

^2^ Chemical Sciences Division, Oak Ridge National Laboratory, Oak Ridge, TN, 37830

^3^ Environmental Sciences Division, Oak Ridge National Laboratory, Oak Ridge, TN, 37830

^*^Corresponding Author: Dr. Robert L. Hettich

P.O. Box 2008, Oak Ridge National Laboratory, Oak Ridge, TN, 37831-6131

Tel: +1 865 574 4968

E-mail: [hettichrl@ornl.gov](mailto:hettichrl@ornl.gov)

Supporting Information

**Table of Contents**

Materials and Methods2

Chemicals2

Nano-LC-MS/MS Analyses......................................................................................................2

Data Extraction and Processing................................................................................................3

Supplementary Figures5

Supplementary Tables24

Supplementary References31

**Materials and Methods**

**Chemicals.** Acetonitrile (ACN), methanol (MeOH), isopropyl alcohol (IPA) and water (H_2_O), all degassed and LC/MS-grade, were purchased from EMD Millipore (Billerica, MA, USA). Mobile phase (MP) additives including ammonium acetate (NH_4_Ac), ammonium hydroxide (NH_4_­OH), and formic acid (FA), were purchased from Sigma-Aldrich. Authentic standards (> 98 % purity) representing a range of LMW organic compounds (Table S1) were purchased from Fluka-Honeywell Research Chemicals or Sigma-Aldrich. Stock solutions, 1 mmol L^-1^, were dissolved in LC/MS-grade H_2_O and standard curves were prepared by dilution with either ACN or H_2_O, to match starting LC mobile phase conditions. Mixed standard solutions were prepared to final equimolar concentrations of 0.1, 1, 10, and 100 µmol L^-1^. All stock solutions and dilutions were stored at -20 °C until analysis and FA or NH_4_­OH were added immediately prior to analysis.

**Nano-LC-MS/MS Analysis.** The ESI source capillary temperature and voltage were optimized to 225 or 275 °C and 2.2 or 2.8 kV, for negative- or positive-ion mode, respectively. Full precursor (MS^1^) scans were acquired in centroid mode at a resolving power of 30,000 over a mass range of 50 – 1000 *m/z*. Fragmentation data (MS^2^) were collected to provide a third dimension for annotation (RT, MS^1^, and MS^2^) and structural information to help eliminate candidates from multiple database hits. Collision-induced dissociation with He_(g)_ was performed on the top 5 ions for each full scan at 15,000 resolving power, a 2 *m/z* isolation width, and an optimized 30 % normalized collision energy for fragmentation. Monoisotopic precursor ions that were selected for fragmentation were placed on a dynamic exclusion list for two minutes and a charge state rejection of doubly-charged precursors was also enforced to improve detection and isolation of low abundant or coeluting small molecules. Two microscans were averaged for every full MS^1^ and MS^2^ spectrum to help reduce the complexity of the mass spectrum. Mass calibration was performed every two days to control for instrument drift using a mixture of caffeine, MRFA and Ultramark 1621 in ACN, MeOH, and acetic acid for positive-ion mode and a mixture of sodium dodecyl sulfate, sodium taurocholate, and Ultramark 1621 in ACN, MeOH, and acetic acid for negative-ion mode (Pierce, ThermoFisher Scientific).

**Data Extraction and Processing.** *Artifact removal and data filtering strategy.* In untargeted analyses, it is important to be able to detect as many small, but real analyte signals, and to remove as much noise or background signal as possible. Here, differentiating between true and false signals was accomplished by first optimizing three parameters in the MZmine2 peak extraction algorithm—minimum peak height (intensity > 1.0E4), MS^1^ tolerance (+/- 10 ppm) and RT window (+/- 1 min) for the corresponding MS^2^ spectrum. Peak detection and module parameters were optimized by manually inspecting the accuracy of peak assignment using the internal standards.^1^ Second, by including controls and daily technical blanks, any artifact signals (intensity > 1.0E5) that originated from sample collection, preparation, or analysis (i.e. extraction leachates, solvent contaminants, column background) were easily identified and manually removed, decreasing the false discovery rate in the technique.

Precursor ions that were selected for fragmentation were identified with the MS/MS peaklist builder (+/- 0.005 *m/z* or 10 ppm MS^1^ window) followed by the peak extender module which searches for data points in both directions of the apex RT (MS^1^ mass tolerance +/- 10 ppm, intensity > 1.0E4). Isotopic peaks were then removed with the isotopic peaks grouper module using a +/- 0.001 *m/z* and 1 min RT tolerance. During the ESI process, in-source fragmentation can occur, along with the formation of non-proton adducts or complexes with Na^+^, K^+^, or NH_4_^+^ for example, that coelute with analytes of interest. In this study, adducts, fragments, and complexes were identified in MZmine2 by their accurate mass difference (+/- 5 ppm) and matching RT (+/- 1 min) to the molecular ion. To help reduce any mass shifts that would impact annotation, but include features whose RTs had shifted slightly between extraction replicates, peaks from the same chromatographic phase and ionization mode were aligned (+/- 5 ppm, +/- 4 min RT) based on 10 iterations and a > 80 % match score using the nonlinear, random sample consensus (RANSAC) algorithm.^2-3^ Integrated LC peak areas were obtained from the aligned extracted ion chromatograms and normalized to the internal standards using a ratio factor determined with the standard compound normalizer module. Peak areas were log_2_-transformed to rescale the dataset for statistical analyses, standardized to the dry weight of soil extracted, and normalized by LOESS and median-centering adjustments across the global dataset within the freely-available InfernoRDN software package.^4^

*Statistical Analyses.* Principal component analyses and two-way hierarchical clustering (heatmaps) were used to examine abundance differences between extraction replicates and core depths, and were conducted using InfernoRDN and Perseus^5^. Student’s t-test was used to perform pairwise comparisons between LWM DOM abundances at each depth to identify the features that varied significantly (log_2_ fold change > 1.5, p-value < 0.05) with depth. Features having a null abundance value in their triplicate were imputed with random numbers from a normal distribution. The mean and standard deviation were optimized to simulate abundance values below the noise level (width = 0.3, shift = 2.5).

**Supplementary Figures**

Figure S1: Signal response curves for equimolar mixed standard (a) neat and (b) after being spiked into and then extracted from Arctic soil, detected and quantified by nano-HILIC/MS in positive-ion mode, with a 20-min gradient and 1 µL injection. The average R^2^ values across the standards was 0.9946 and 0.9924 for (a) and (b), respectively. Axes shown in log scale for clarity.

(a)


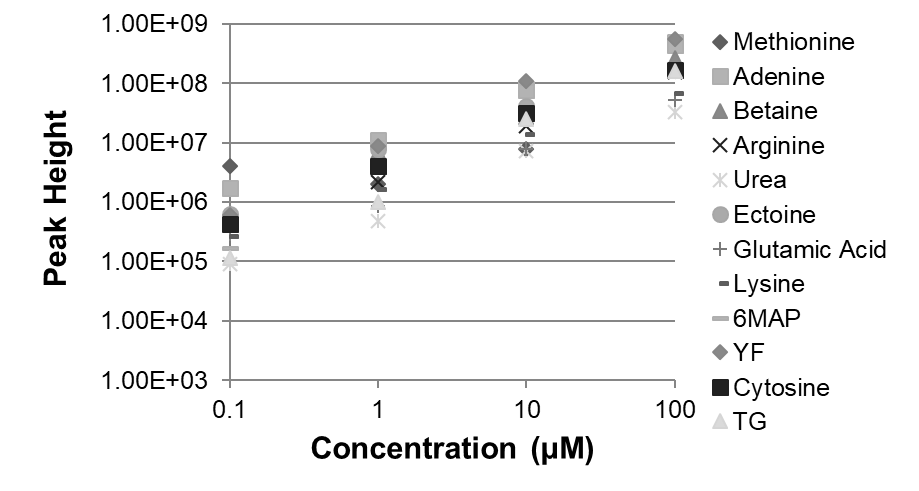


(b)


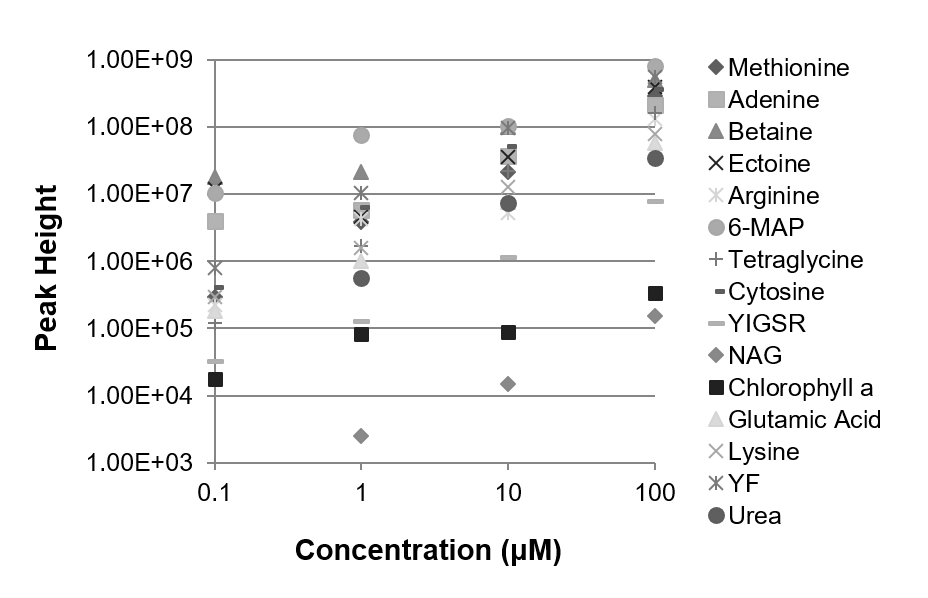


Figure S2: Normalized extracted ion chromatograms (XICs), prior to RT alignment, for the internal standard, 6-methylaminopurine riboside (6-MAP), extracted from nine Arctic soil samples, and detected in positive-ion mode as [M+H]^+^ at 282.1186 *m/*z on the nano-ZIC-pHILIC column.


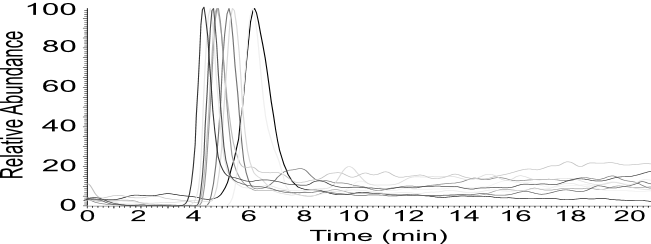


Figure S3: Integrated XIC peak areas for internal standards spiked into and extracted from soil (10 µM), 6-MAP in positive-ion mode (top) and adenosine in negative-ion mode (bottom) detected in triplicate soil water extracts on the HILIC (left) and RP (right) columns. The CV % for each triplicate is also reported (inset).


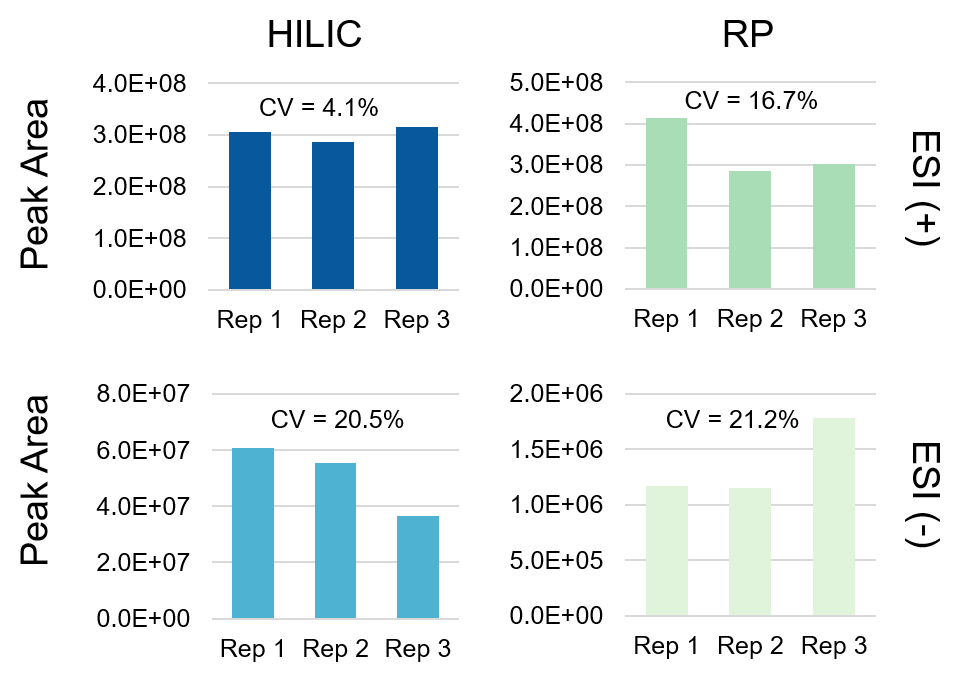


Figure S4: Scatter plots of the features detected (intensity >5.0E4, +/- 0.005 *m/z*) in a single soil water extract and the elution profiles for HILIC (top) and RP (bottom) in ESI (-)-ion mode. Each marker matches to a *m/z* and retention time. The corresponding normalized peak chromatograms are overlaid on top to show a typical elution profile for each LC condition in negative-ion mode.


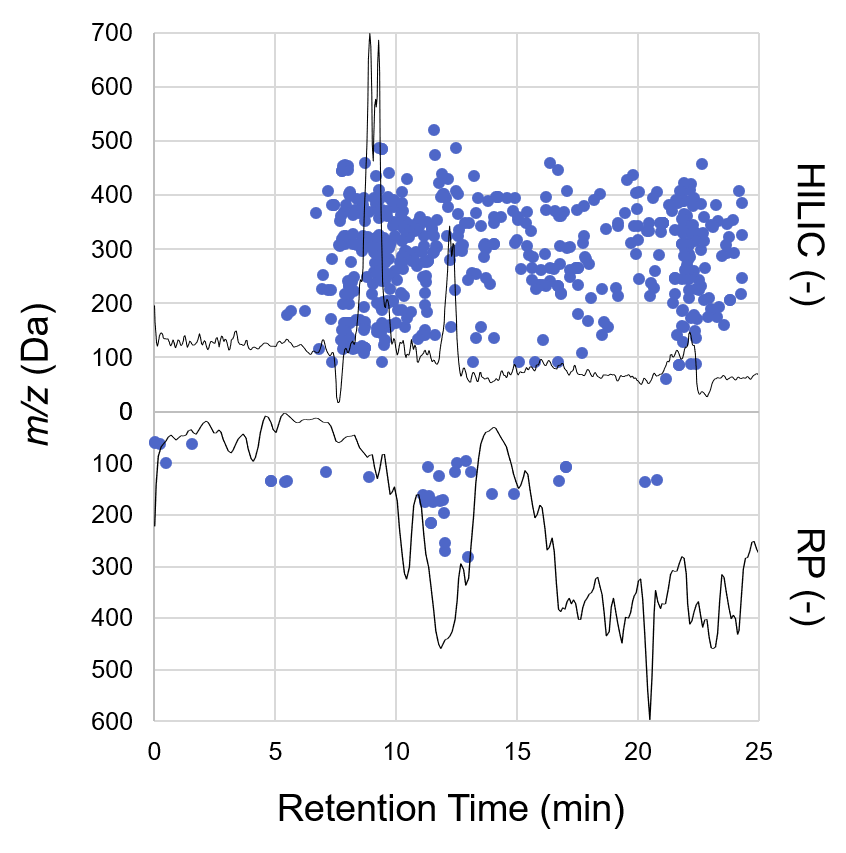


Figure S5: Example of principal component analysis (HILIC (-) analysis) that used a unique identifier and peak areas to analyze the separation between features observed in the 9 soil extracts and 3 controls, demonstrating strong separation between LWM DOM analytes and artifacts.


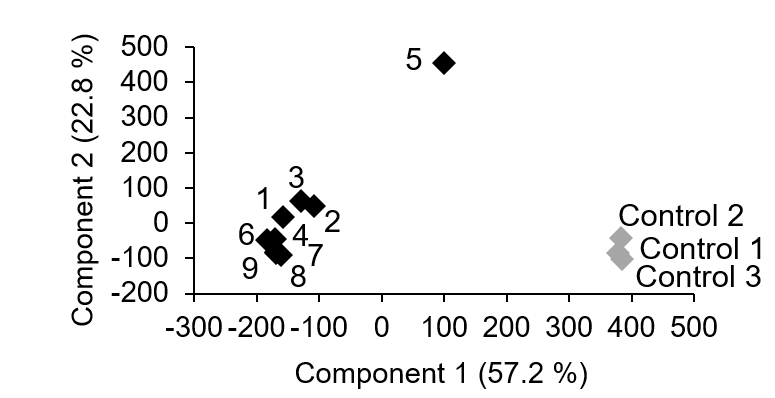


Figure S6: Principal component analysis of high-quality features detected in the 9 soil water extracts by each of the 4 LC-MS conditions evaluated demonstrating complementarity of the techniques: dark blue triangles, HILIC (-); dark green squares, RP (-); light blue diamonds, RP (+); and light green circles, HILIC (+). For more detailed LC-MS conditions, see Table S3.


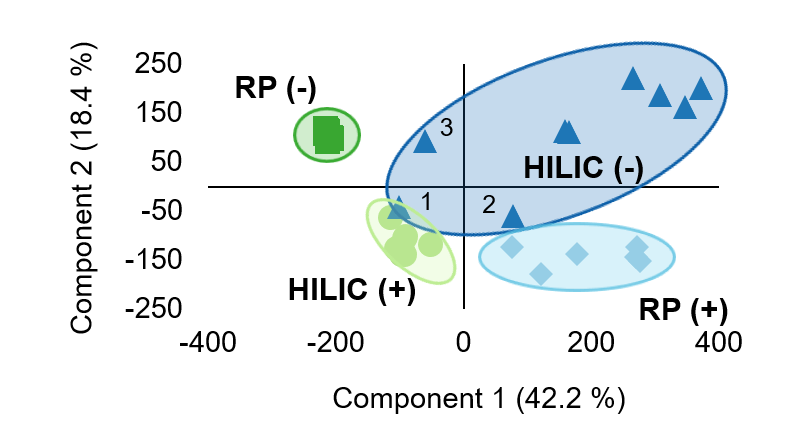


Figure S7: Experimental MS^2^ spectrum for feature detected by HILIC (+) at MS^1^ 116.0705 *m/z*.


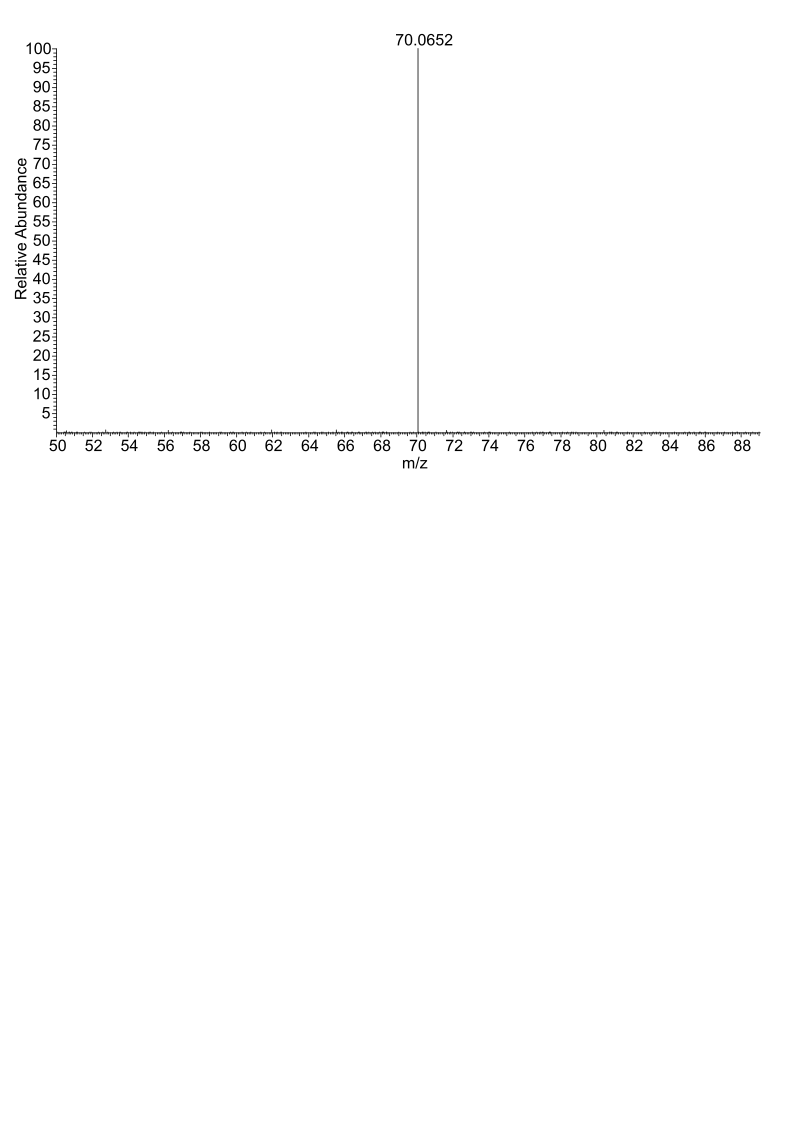


Figure S7b: Corresponding database MS^2^ spectrum for matched standard, proline. Note: Databases where matched compound information were obtained from are listed in Table S4.


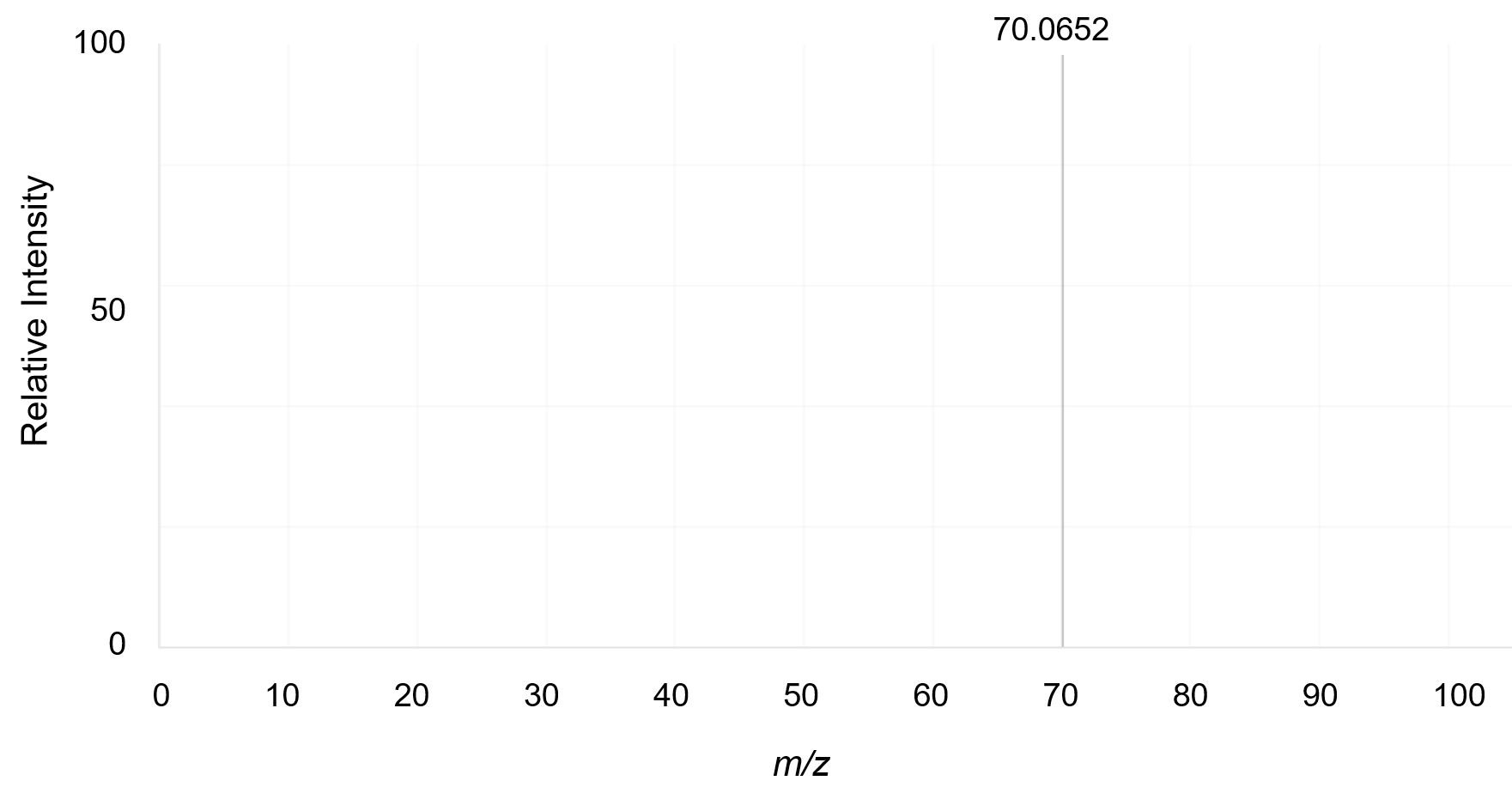


Figure S8: Experimental MS^2^ spectrum for feature detected by HILIC (+) at MS^1^ 120.0807 *m/z.*

**
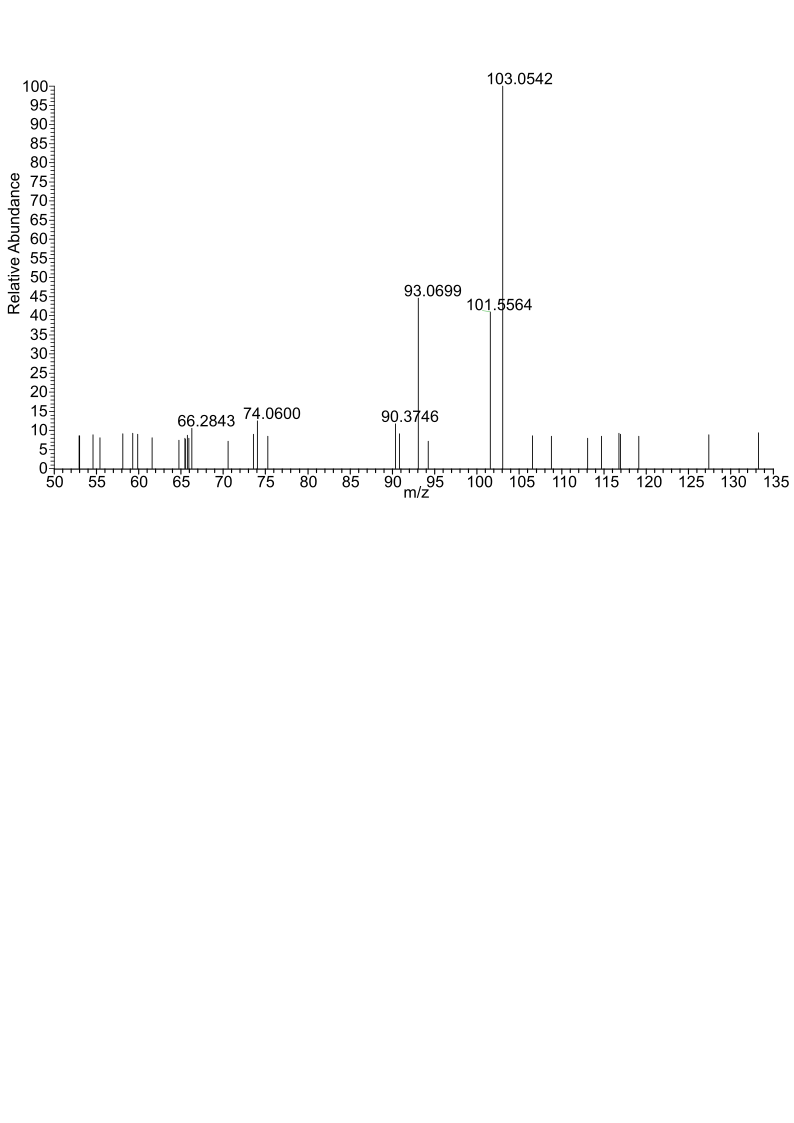
**

Figure S8b: Corresponding database MS^2^ spectrum for matched standard, indoline.

**
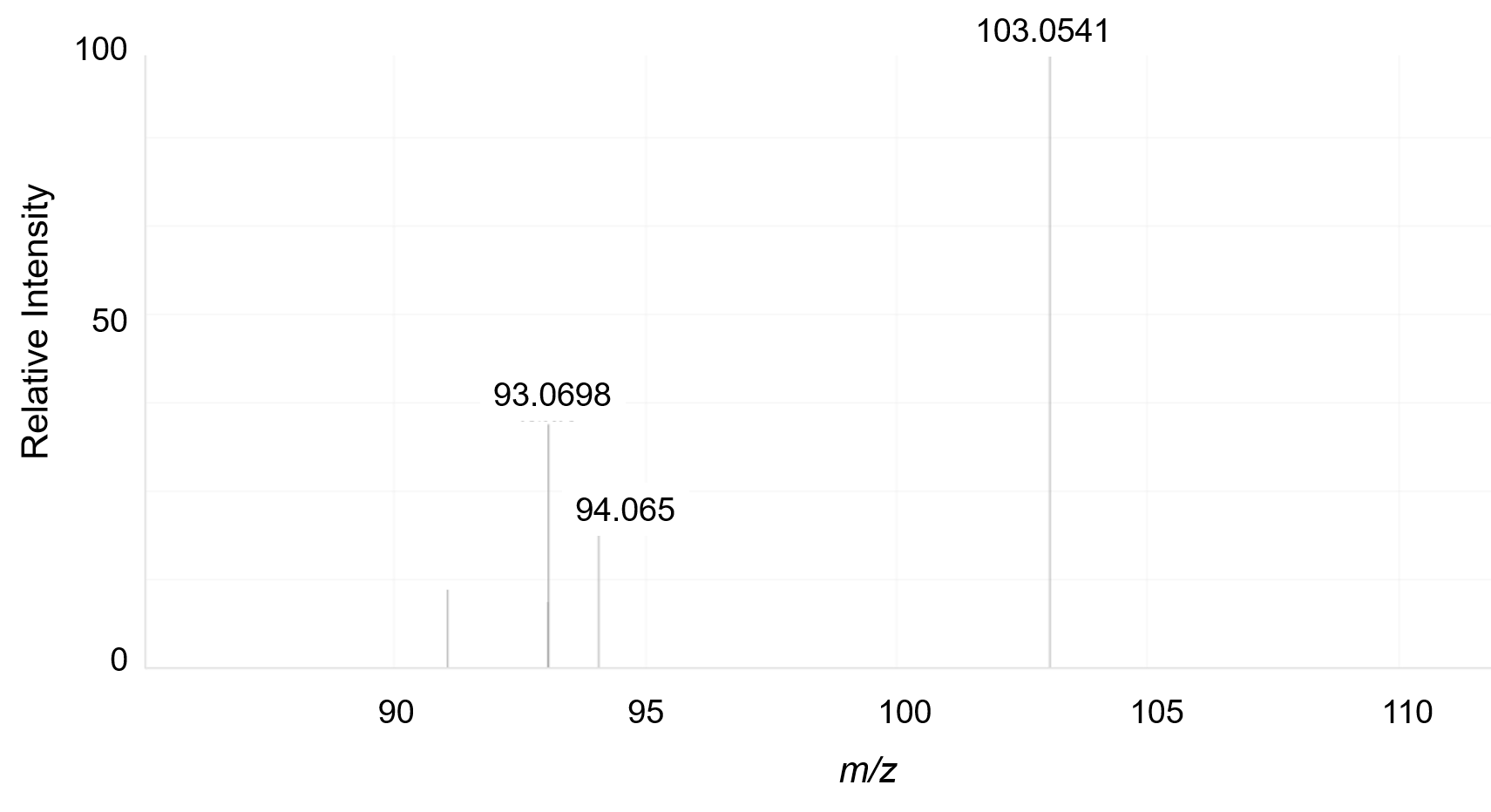
**

Figure S9: Experimental MS^2^ spectrum for feature detected by HILIC (+) at MS^1^ 132.1018 *m/z.*


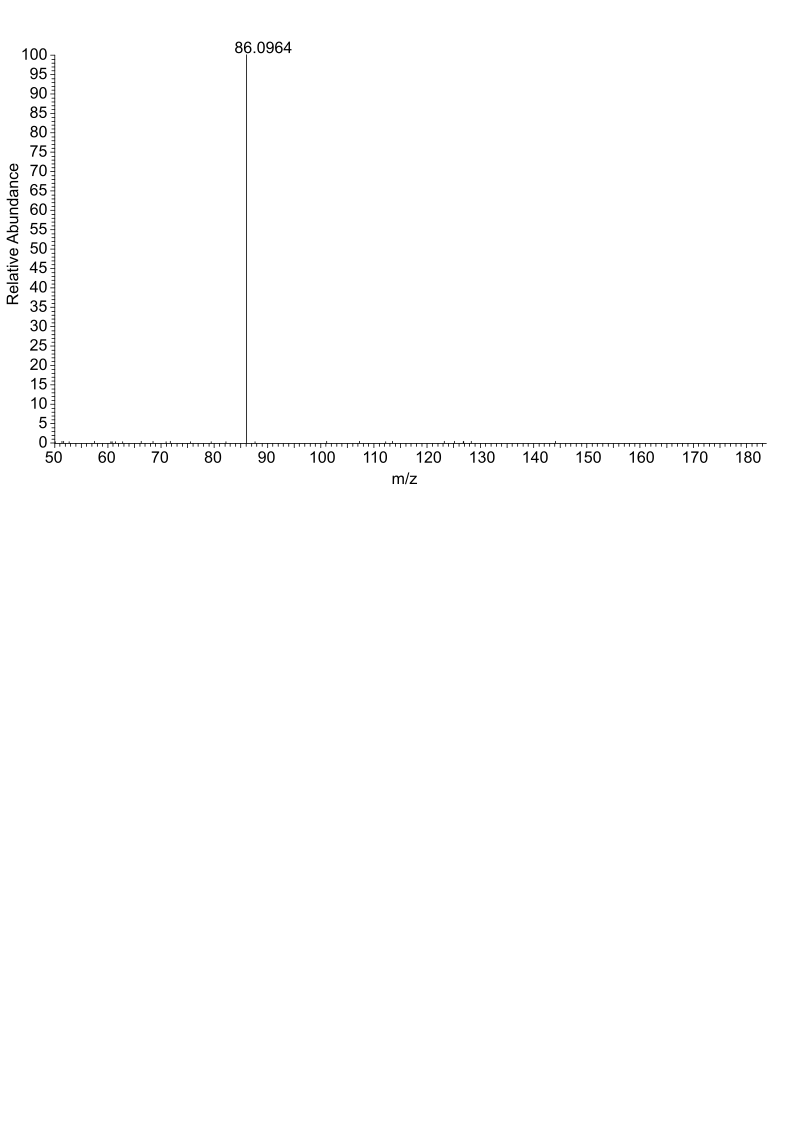


Figure S9b: Corresponding database MS^2^ spectrum for matched standard, alloisoleucine.

**
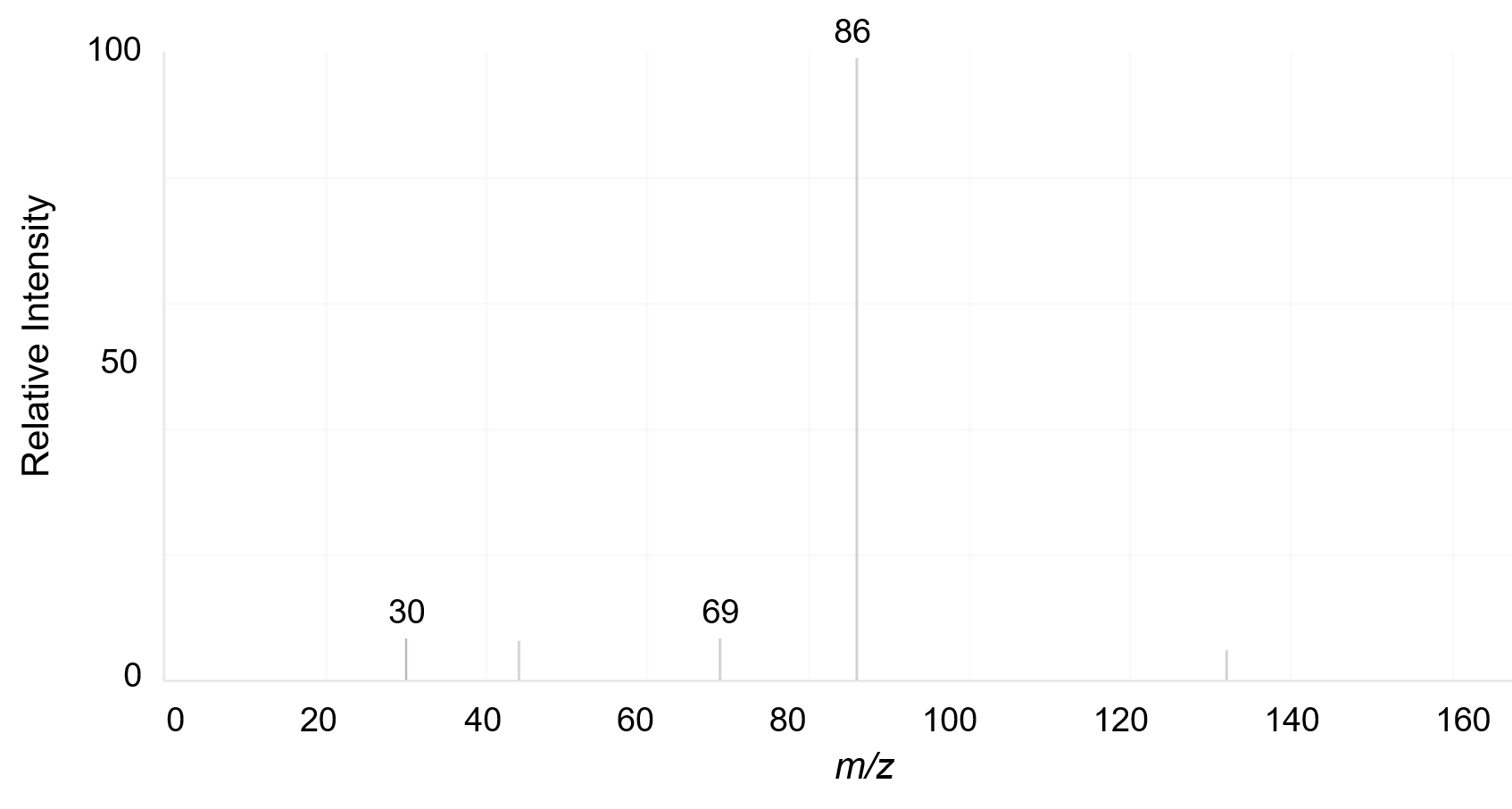
**

Figure S10: Experimental MS^2^ spectrum for feature detected by HILIC (+) at MS^1^ 176.1028 *m/z.*

**
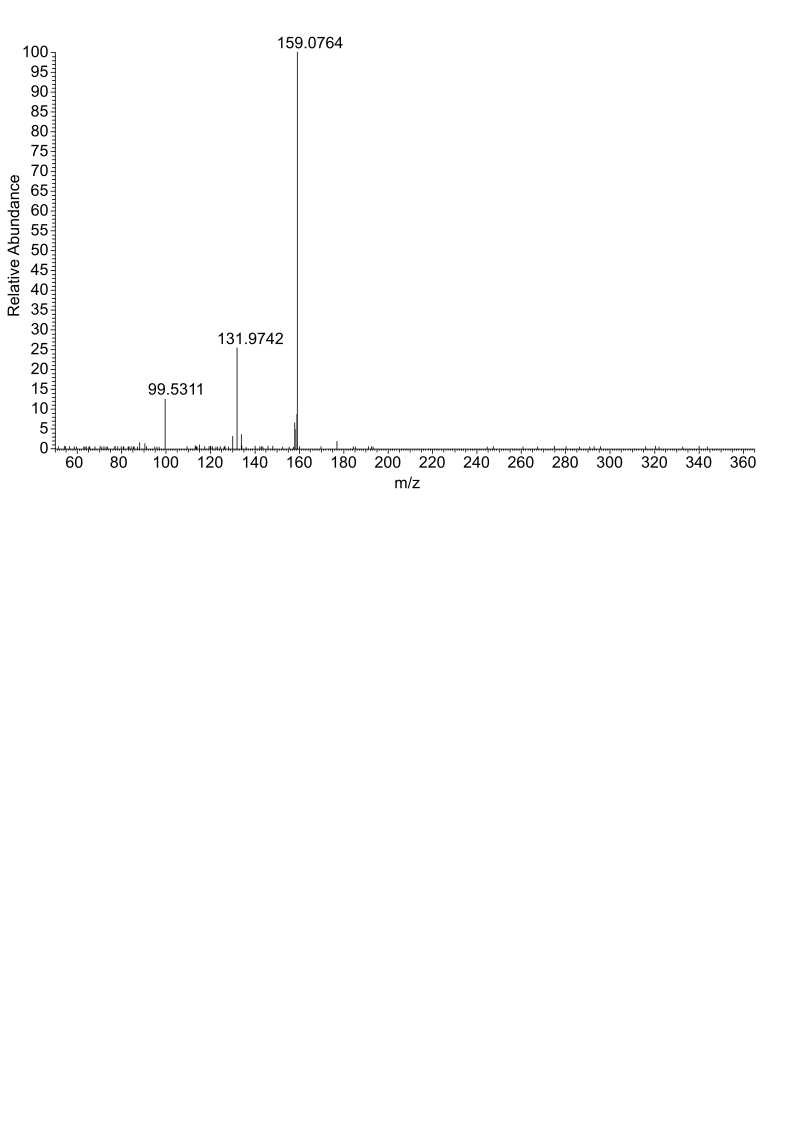
**Figure S10b: Corresponding database MS^2^ spectrum for matched standard at lower CID energy (30 CID), citrulline.

**
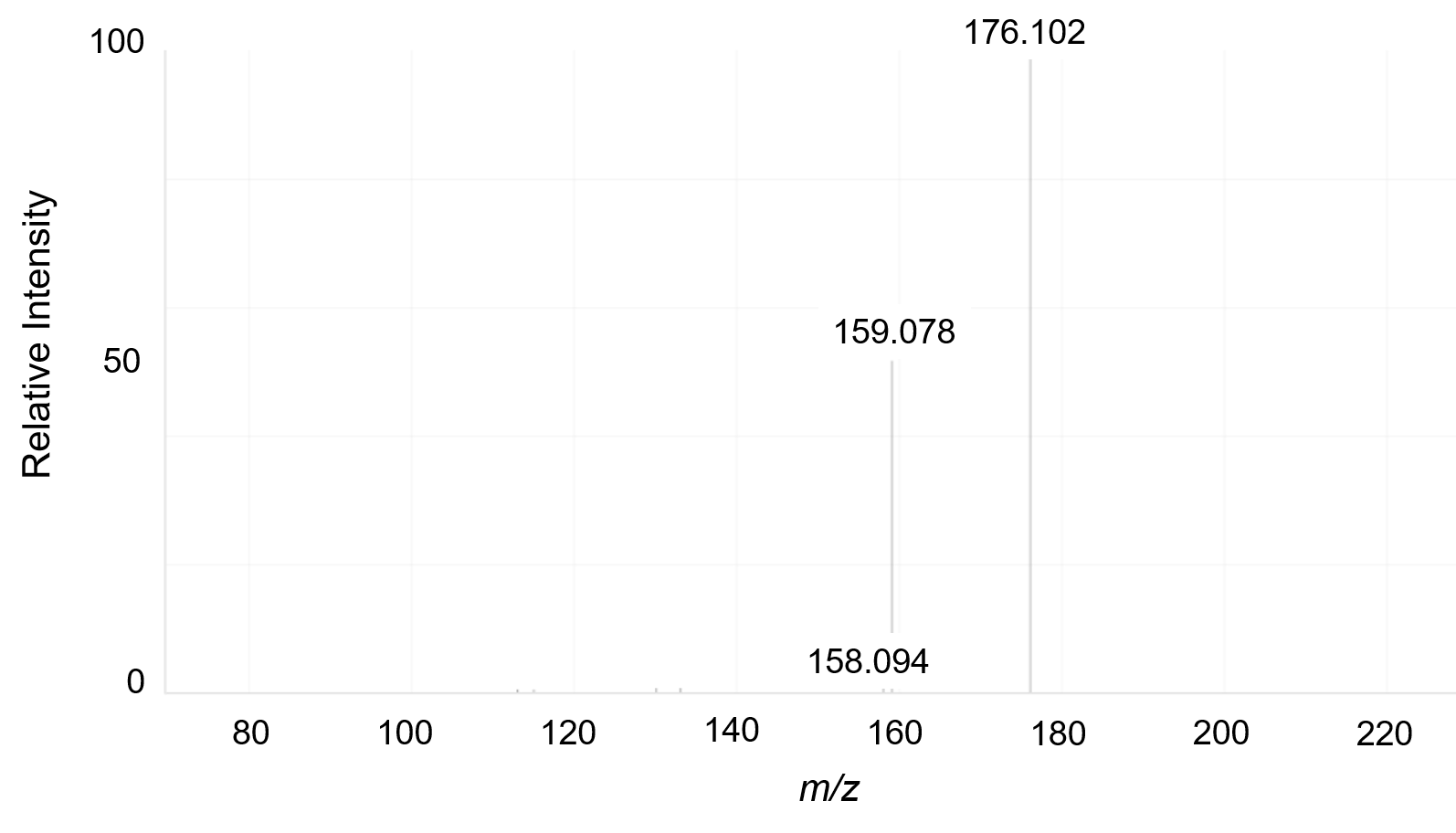
**

Figure S11: Experimental MS^2^ spectrum for feature detected by HILIC (+) at MS^1^ 182.0811 *m/z.*


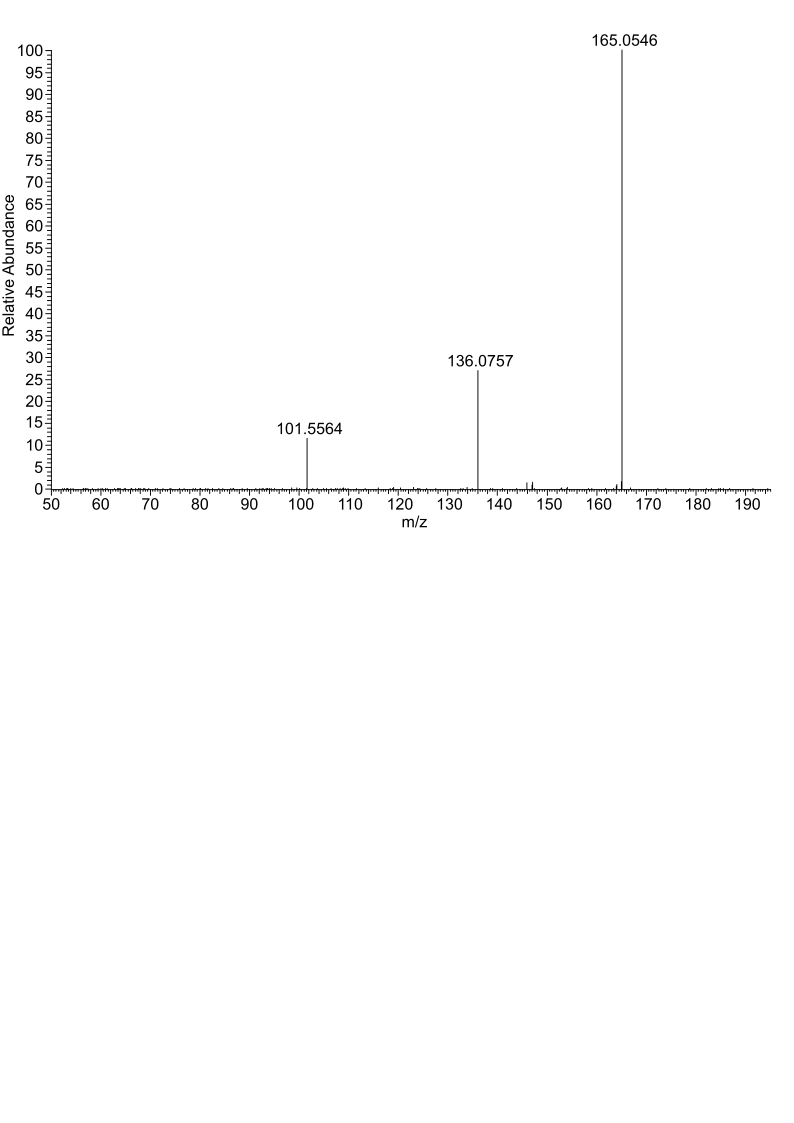


Figure S11b: Corresponding database predicted MS^2^ spectrum for matched compound at higher CID energy (40 CID), beta-tyrosine.


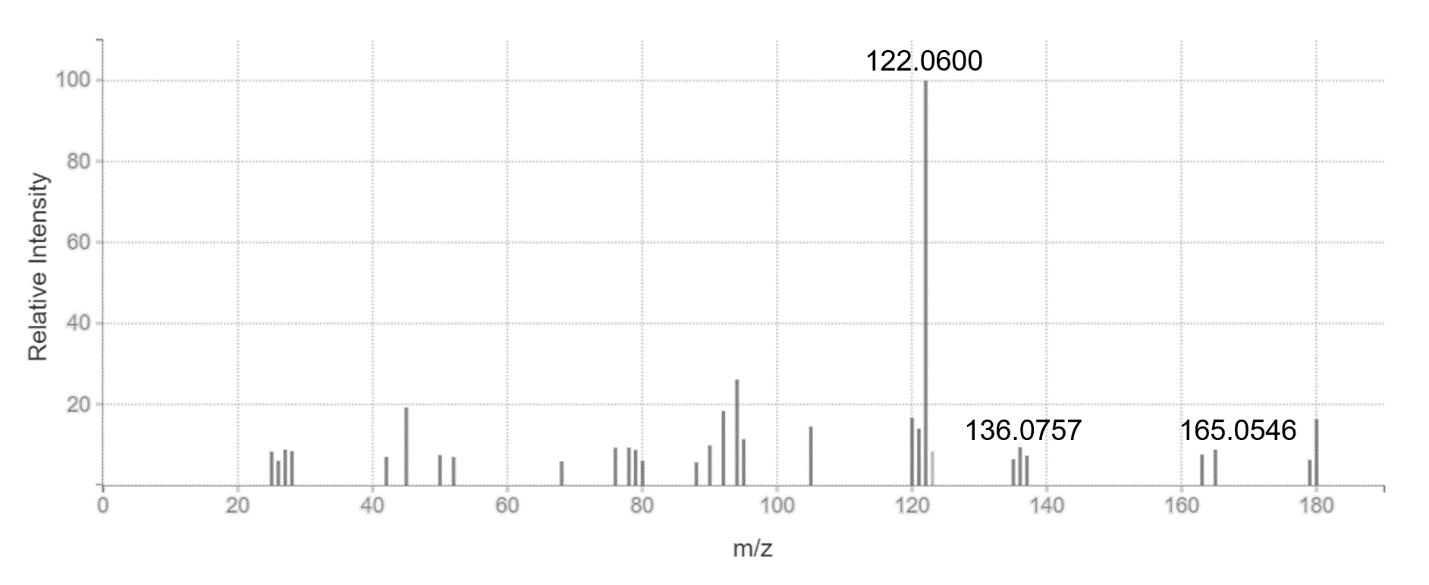


Figure S12: Experimental MS^2^ spectrum for feature detected by HILIC (+) at MS^1^ 188.0705 *m/z.*


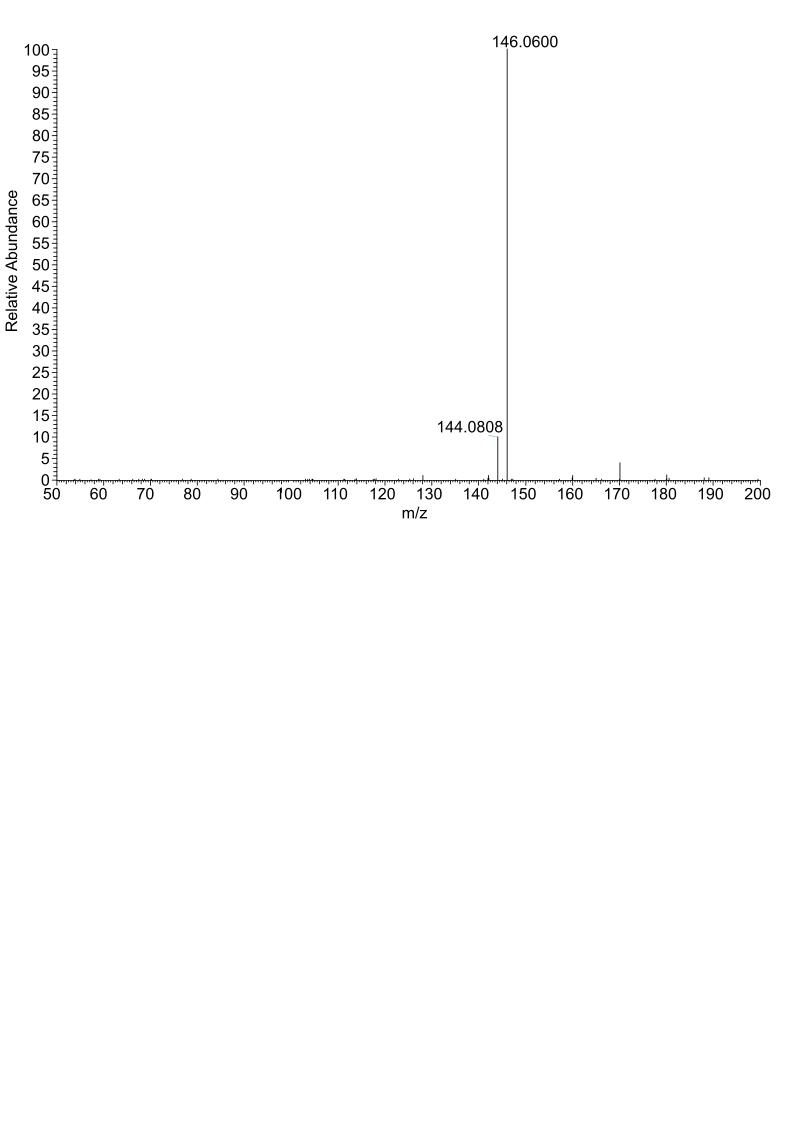


Figure S12b: Corresponding database MS^2^ spectrum for matched compound, N-(2,5-Dihydroxyphenyl) pyridinium standard (predicted MS^2^ at 20 CID energy).


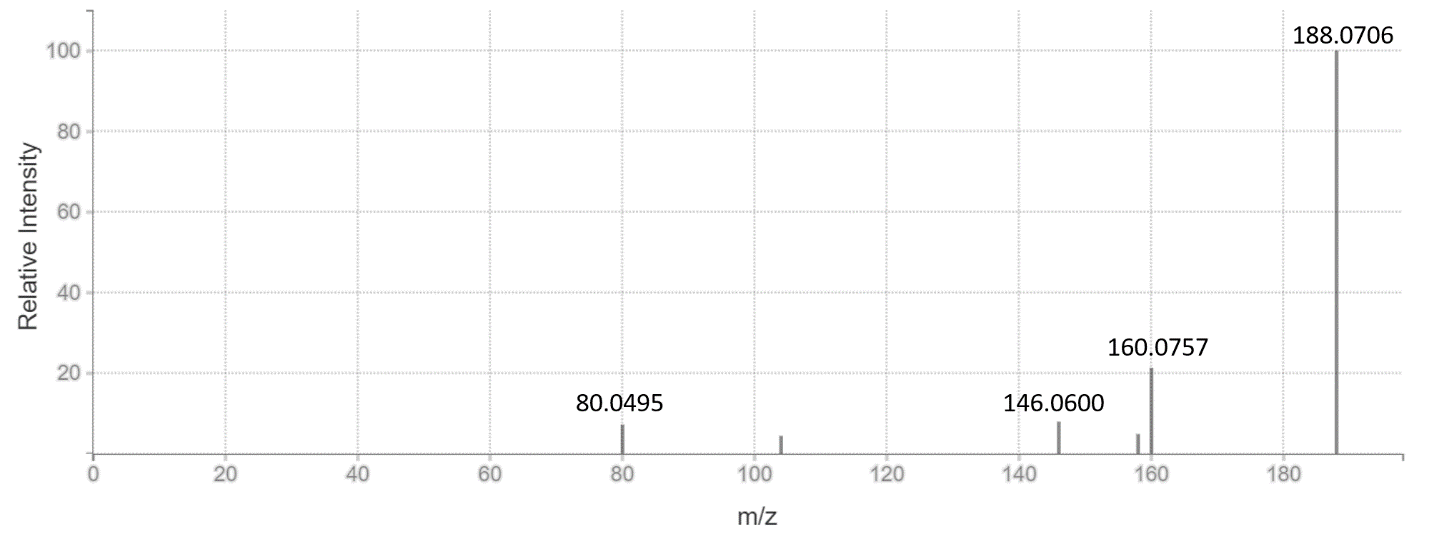


Figure S13: Experimental MS^2^ spectrum for feature detected by HILIC (+) at MS^1^ 220.1178 *m/z.*

**
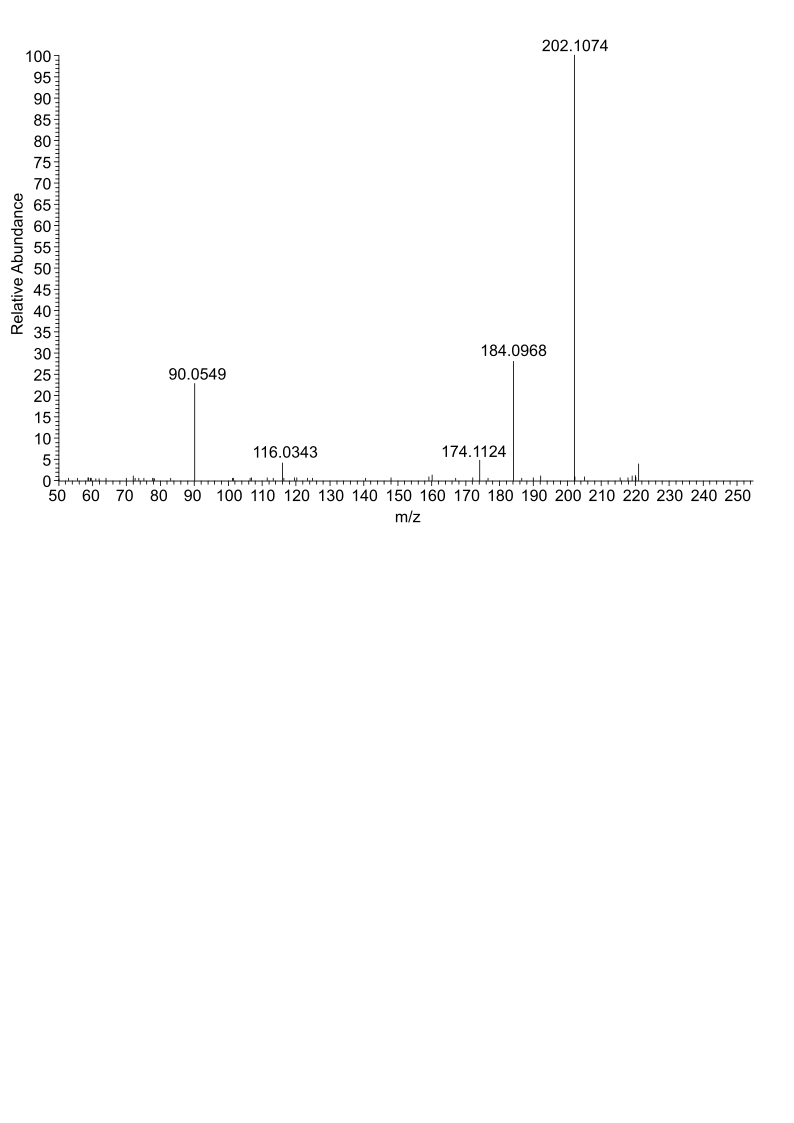
**

Figure S13b: Corresponding database MS^2^ spectrum for matched standard, pantothenic acid.


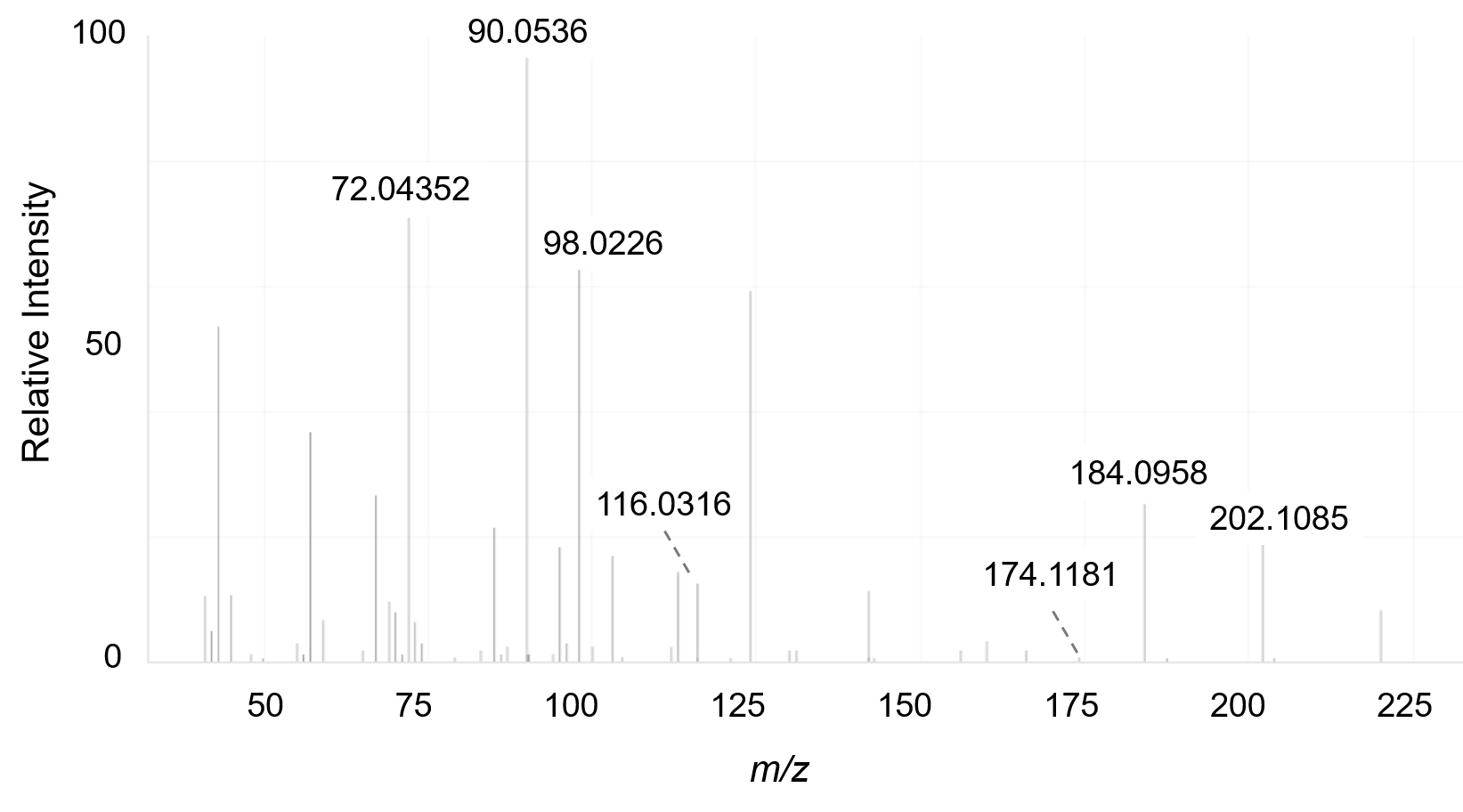


Figure S14: Experimental MS^2^ spectrum for feature detected by HILIC (-) at MS^1^ 219.1021 *m/z.*


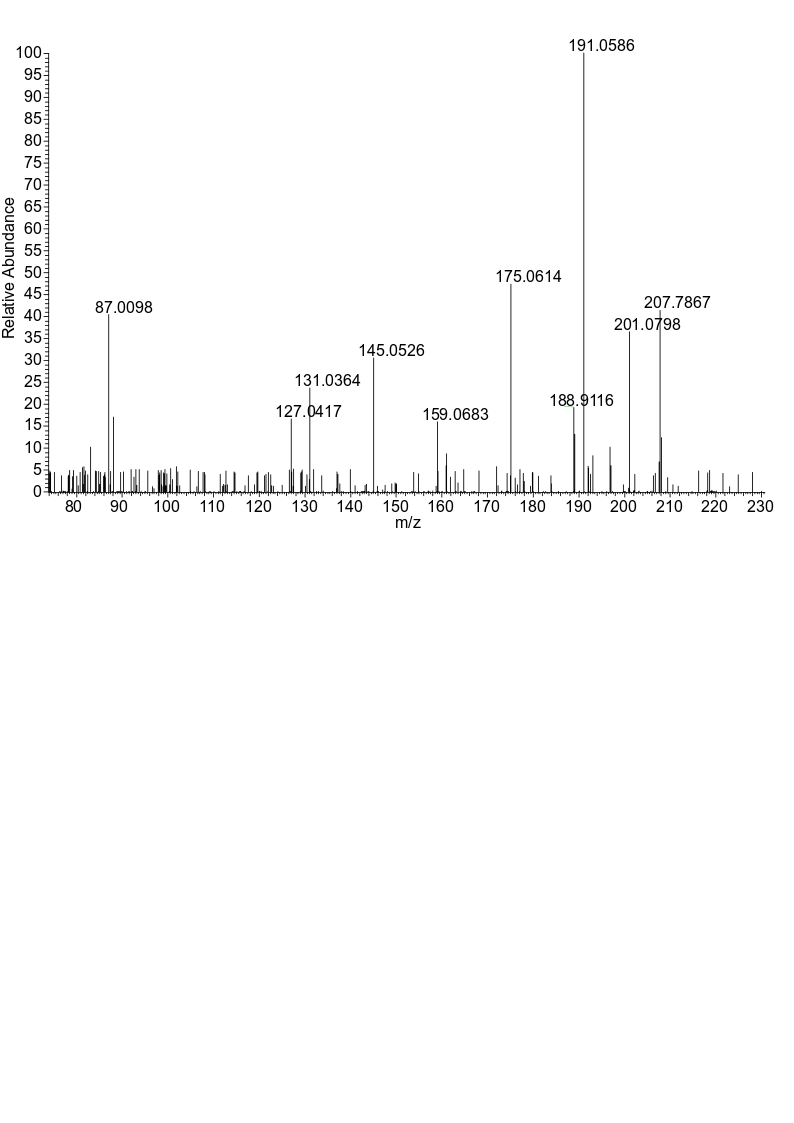


Figure S14b: Corresponding database predicted MS^2^ spectrum for matched compound, ethyl 2-benzylacetoacetate.


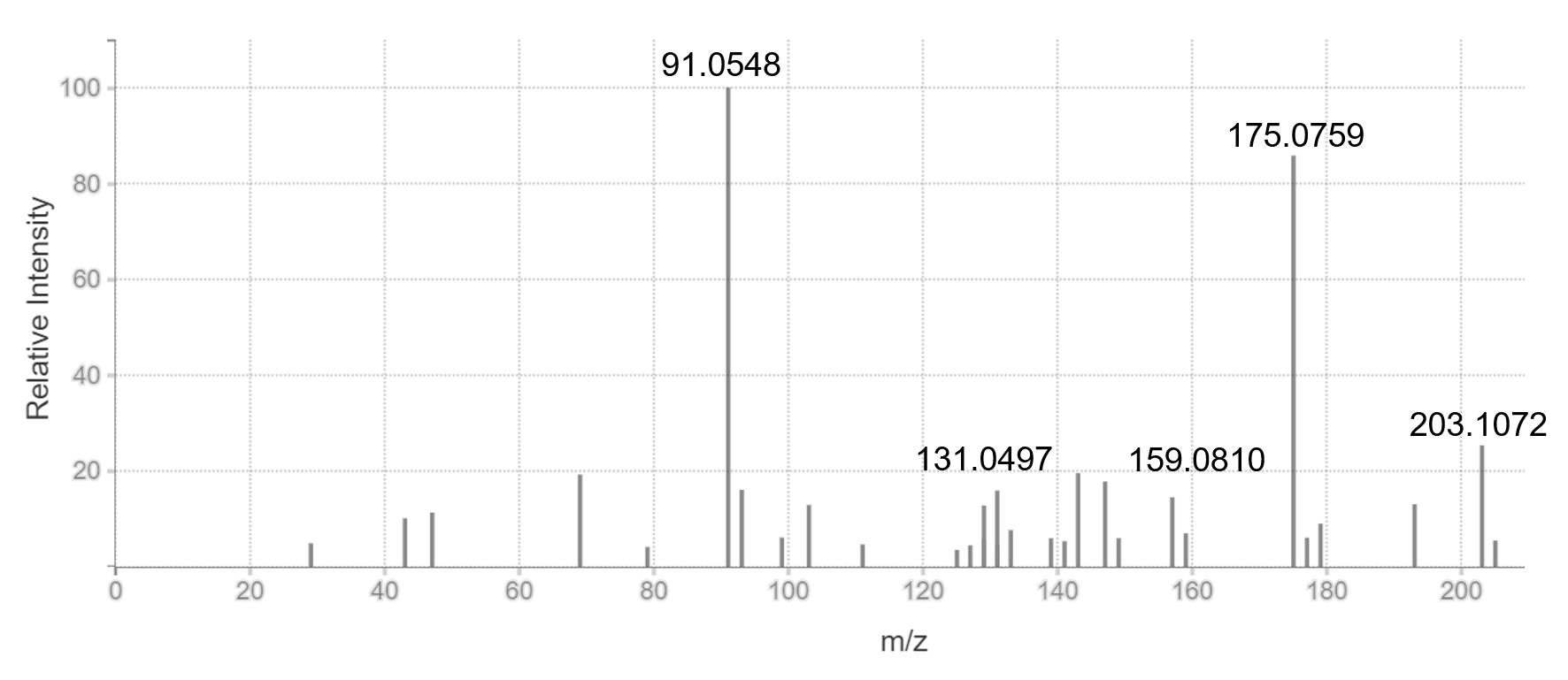


Figure S15: Experimental MS^2^ spectrum for feature detected by HILIC (-) at 227.1074 *m/z.*


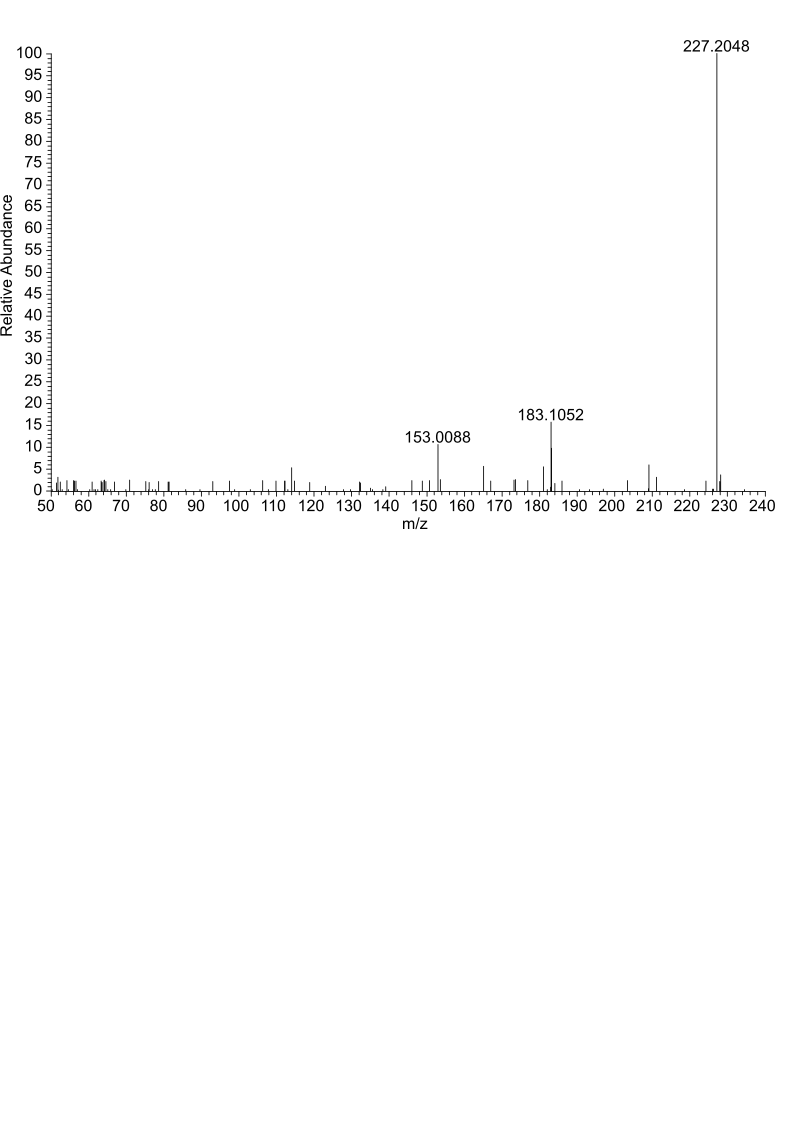


Figure S15b: Corresponding database predicted MS^2^ spectrum for matched compound, pyroglutamylvaline.


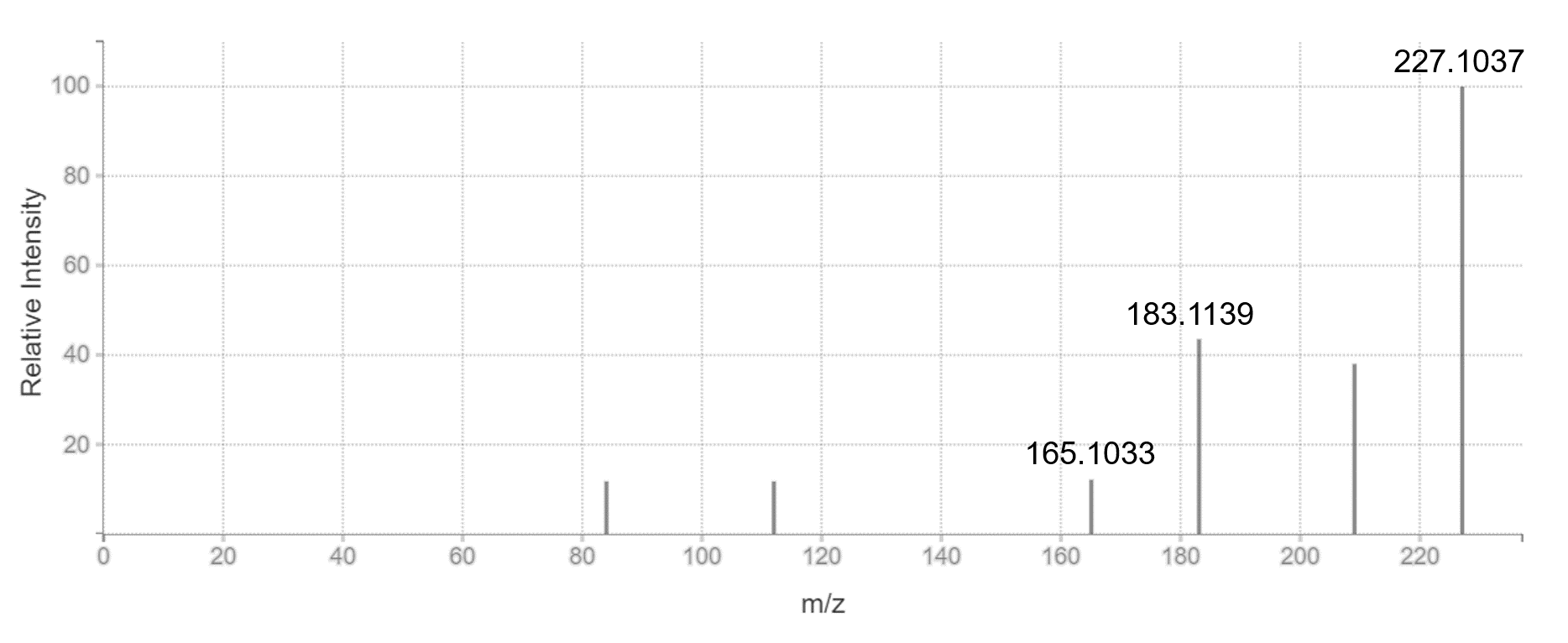


Figure S16: Experimental MS^2^ spectrum for feature detected by HILIC (-) at 229.1239 *m/z.*


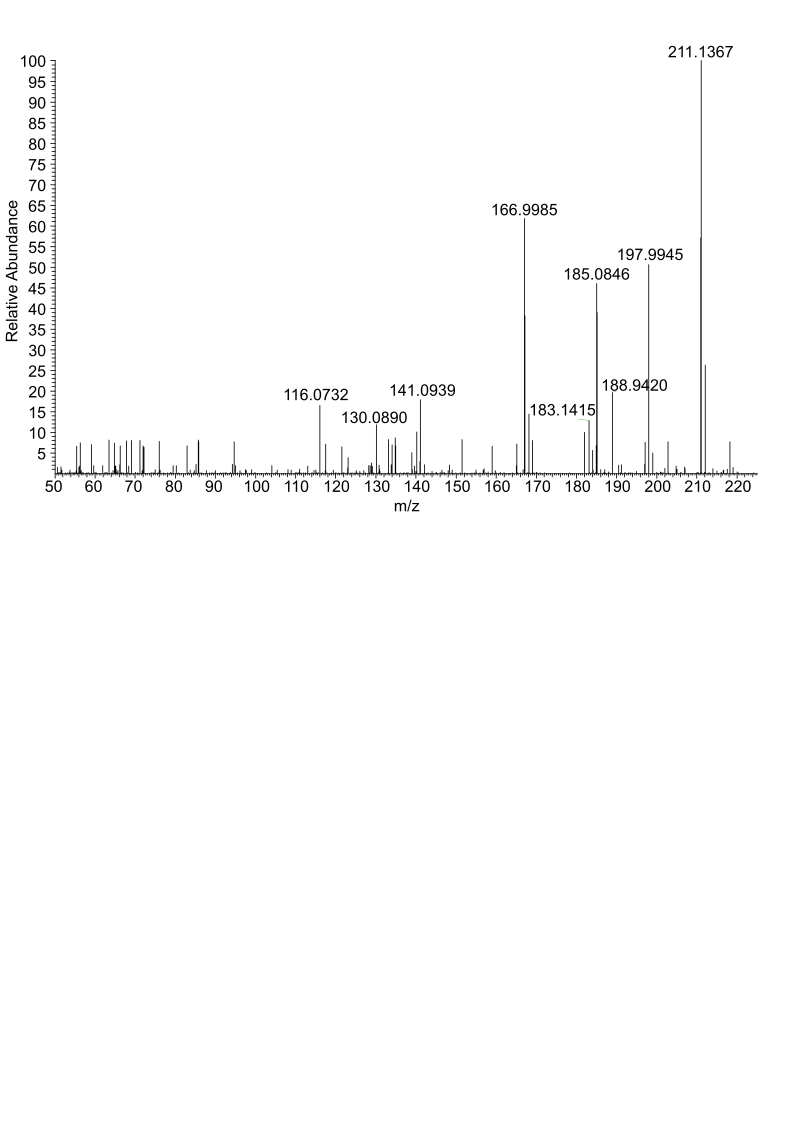


Figure S16b: Corresponding database predicted MS^2^ spectrum for matched compound, 8,12-Epoxy-4(15),7,11-eudesmatrien-1-one.


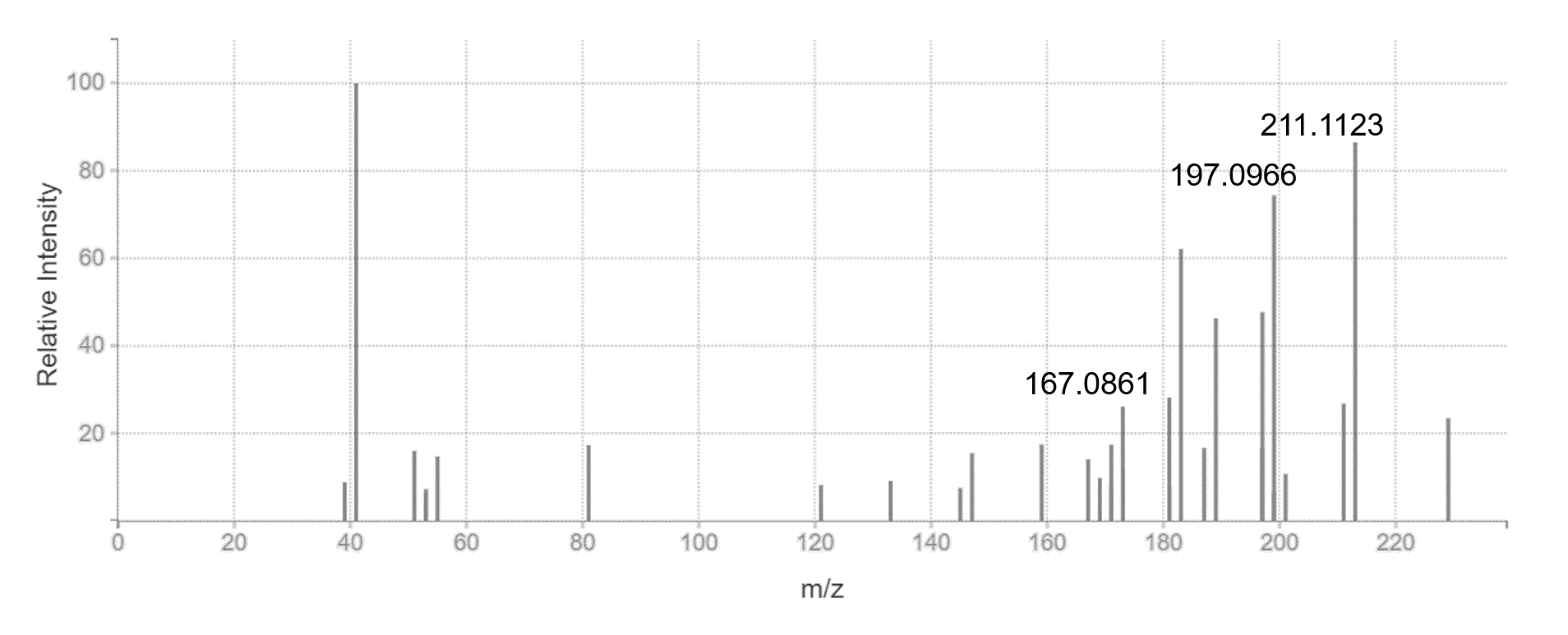


Figure S17: Experimental MS^2^ spectrum for feature detected by HILIC (-) at 293.1442 *m/z.*


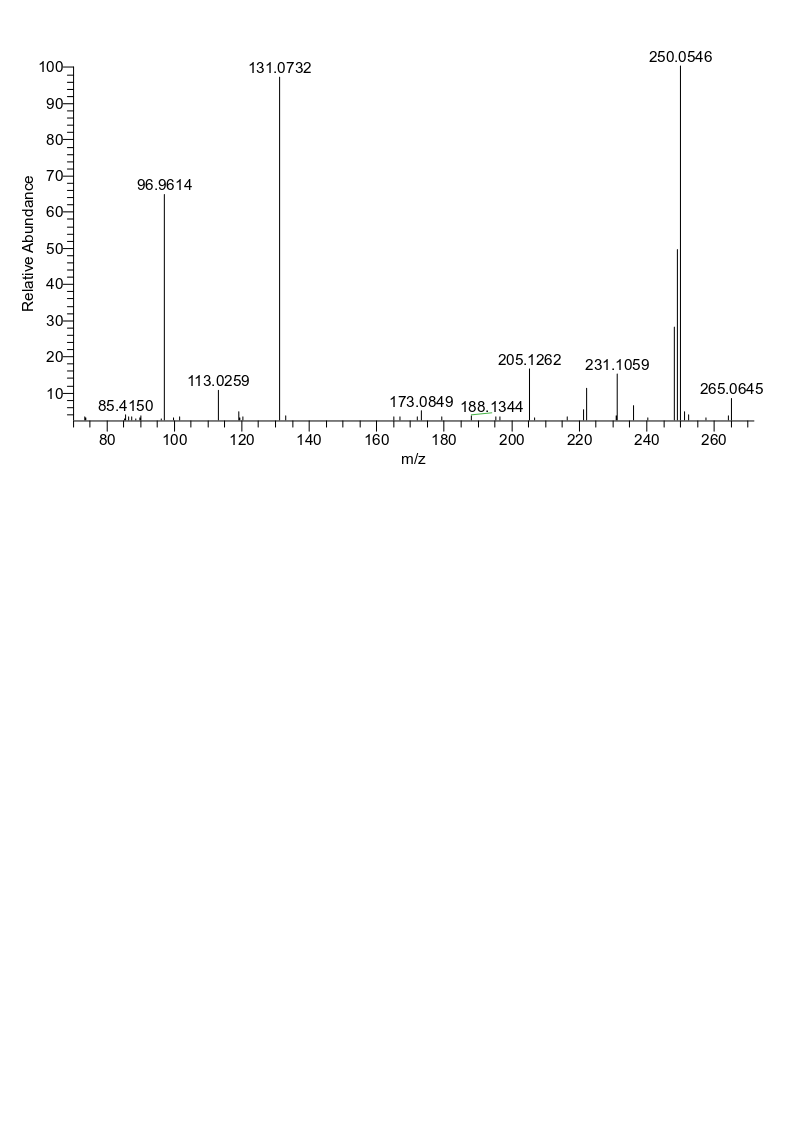


Figure S17b: Corresponding database predicted MS^2^ spectrum for matched compound, heptyl 1-thiohexopyranoside.


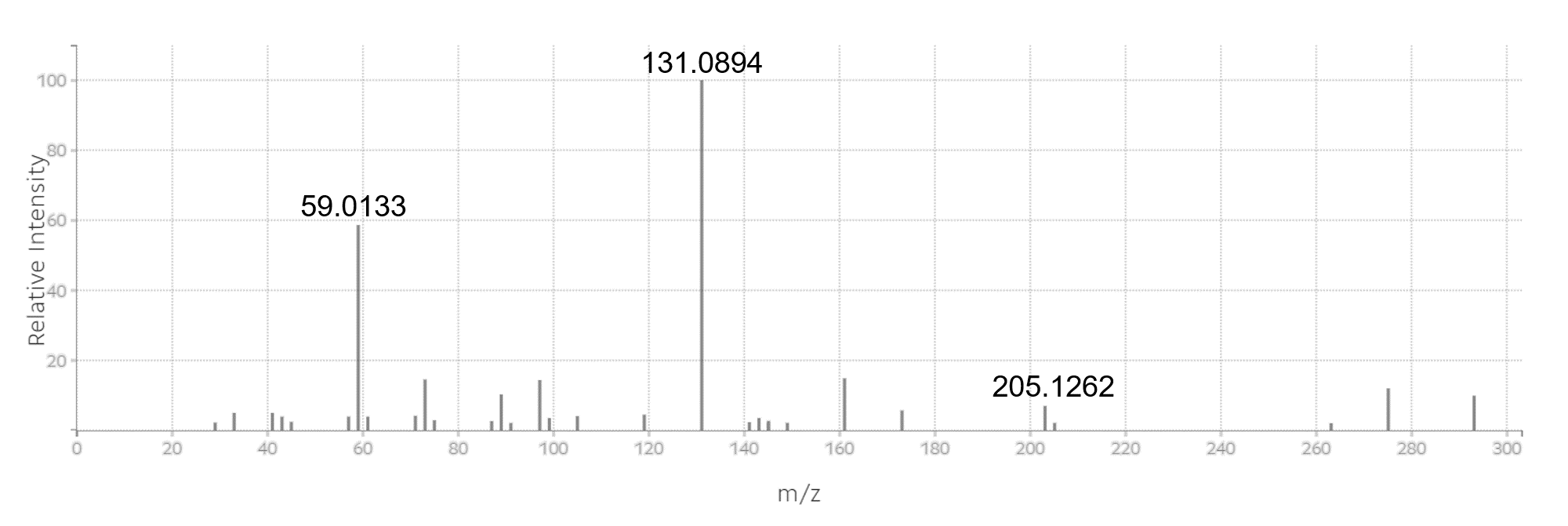


Figure S18: Experimental MS^2^ spectrum for feature detected by HILIC (-) at 457.1309 *m/z.*


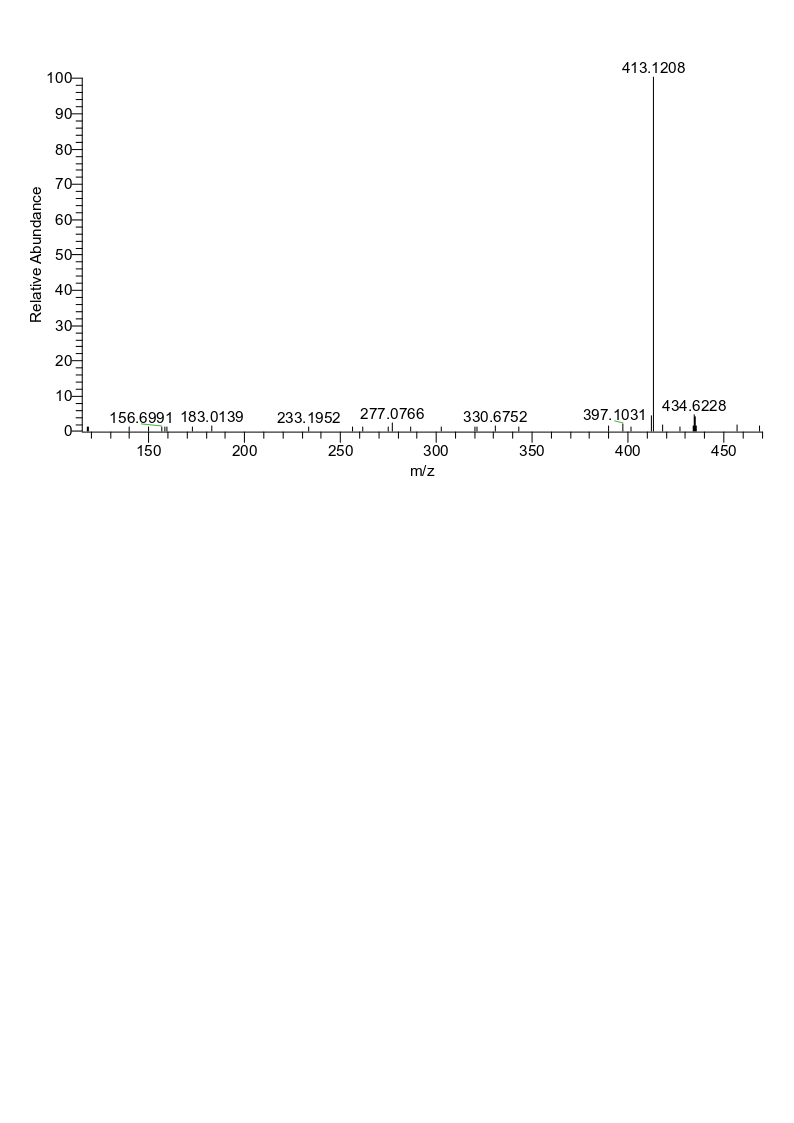


Figure S18b: Corresponding database predicted MS^2^ spectrum for matched compound, a-L-Arabinofuranosyl-(1->3)-b-D-xylopyranosyl-(1->4)-D-xylose at higher CID energy (40 CID).


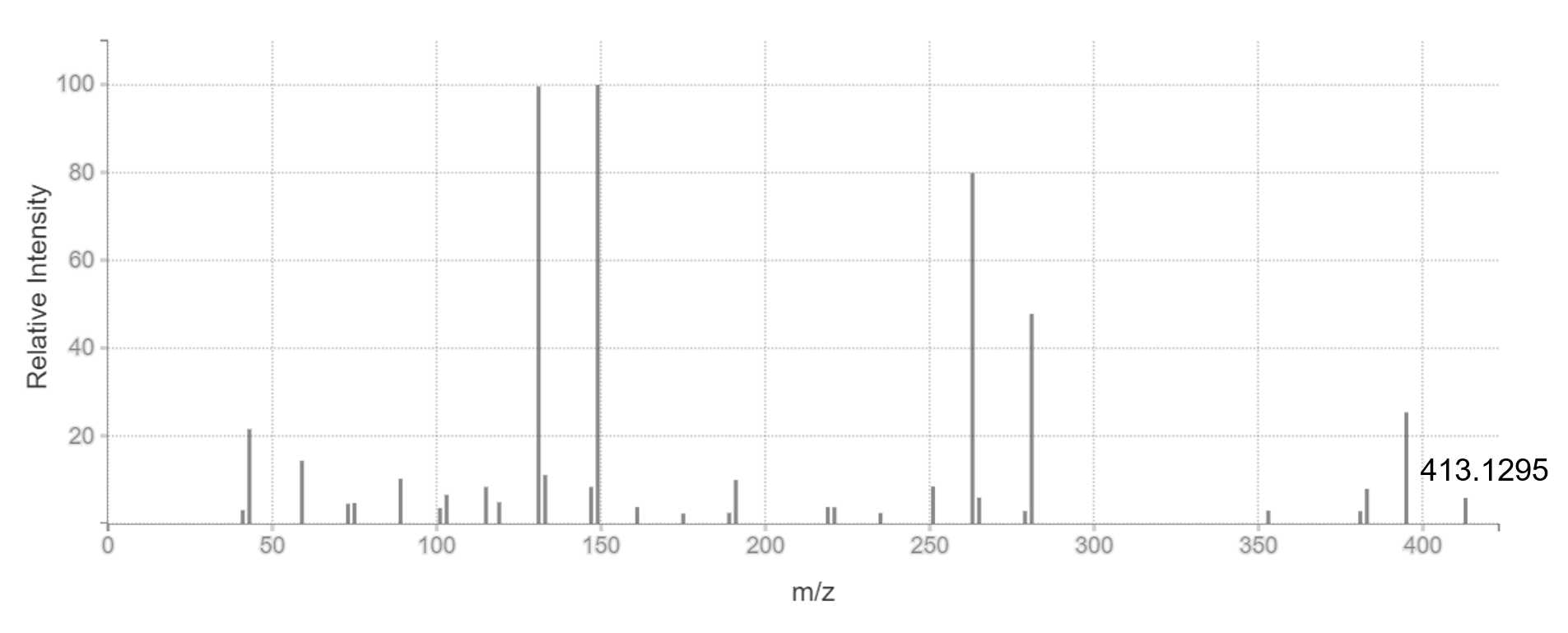


Figure S19: Experimental MS^2^ spectrum for feature detected by RP (+) at 60.0444 *m/z.*

**
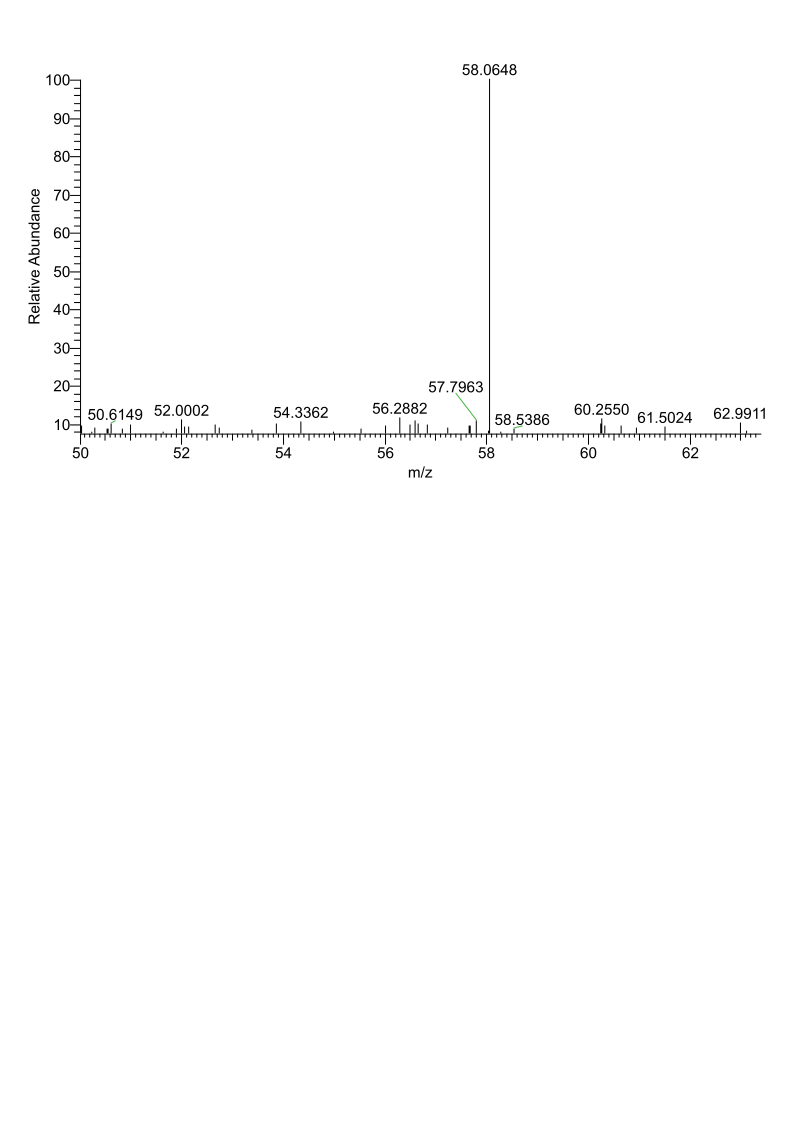
**

Figure S19b: Corresponding database predicted MS^2^ spectrum for matched compound, aminoacetaldehyde.

**
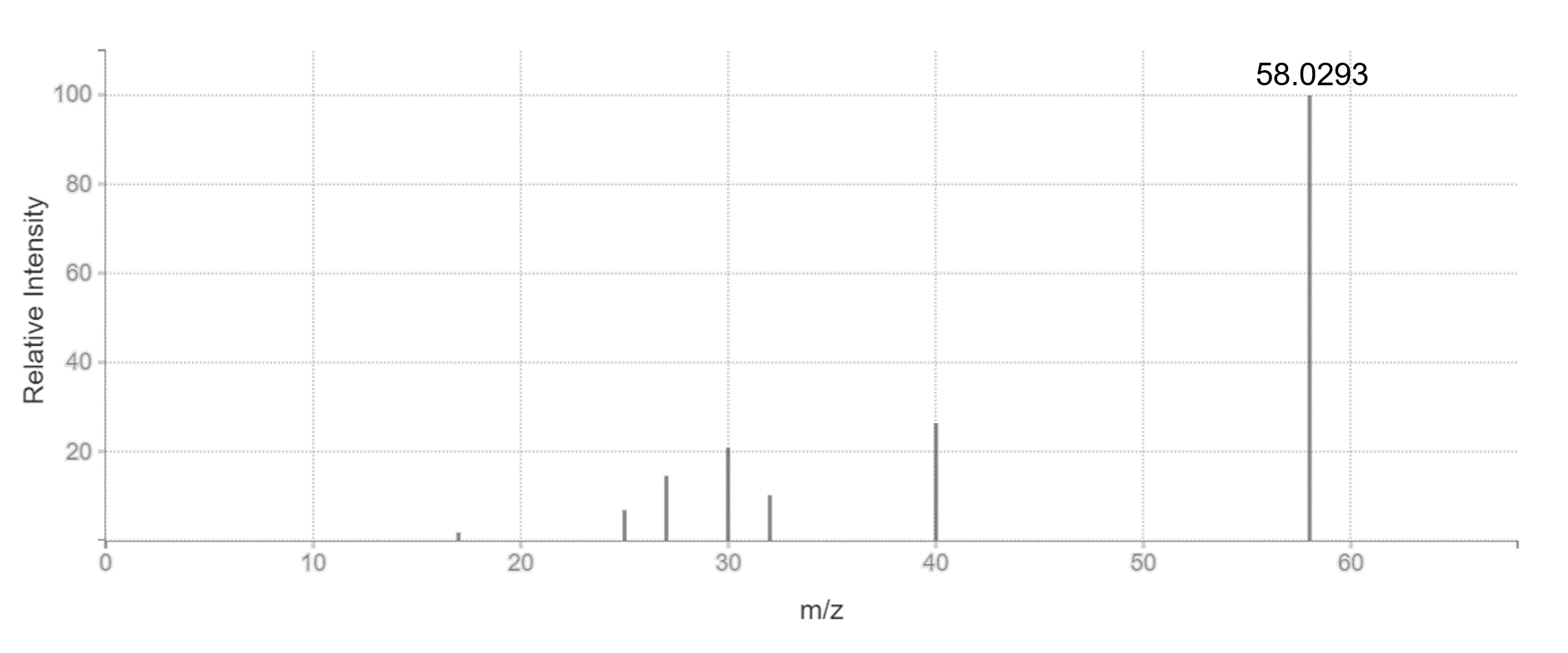
**

Figure S20: Experimental MS^2^ spectrum for feature detected by RP (+) at 101.0709 *m/z.*

**
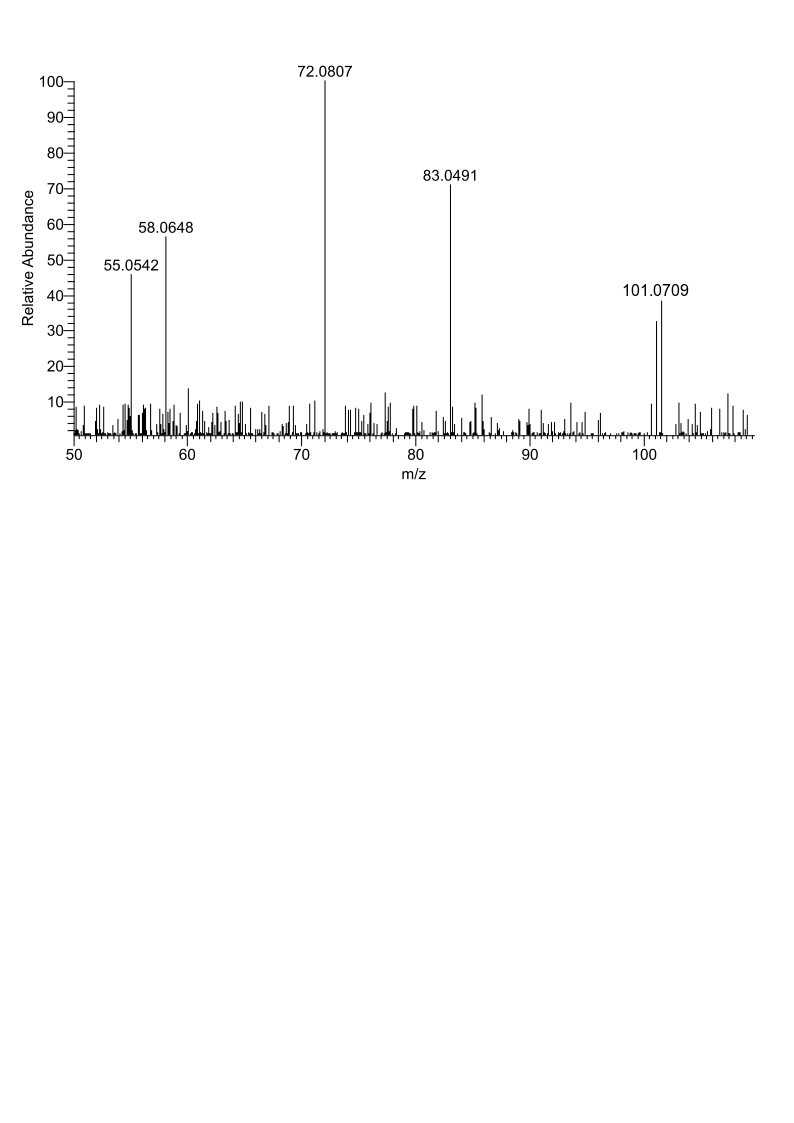
**

Figure S20b: Corresponding database predicted MS^2^ spectrum for matched compound, N-nitroso-pyrrolidine.


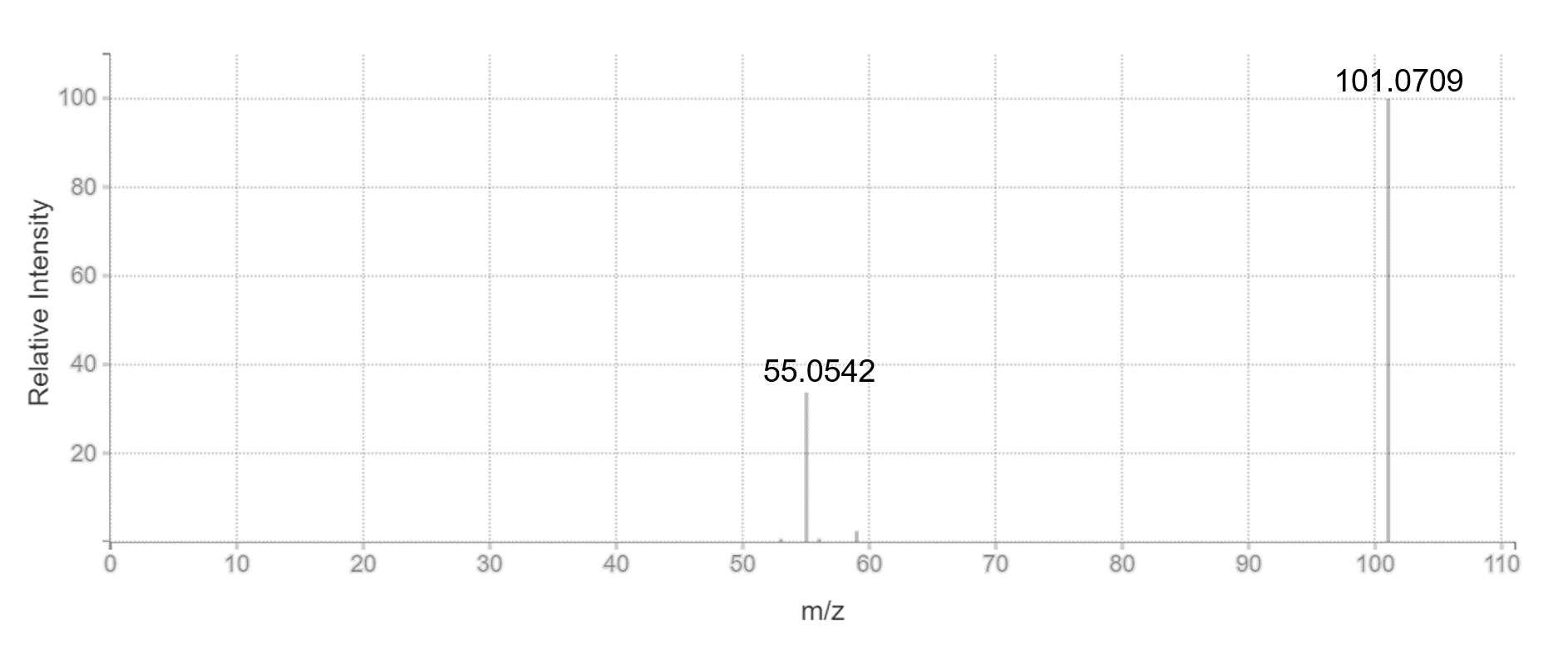
Figure S21: Experimental MS^2^ spectrum for feature detected by RP (+) at 195.0512 *m/z.*


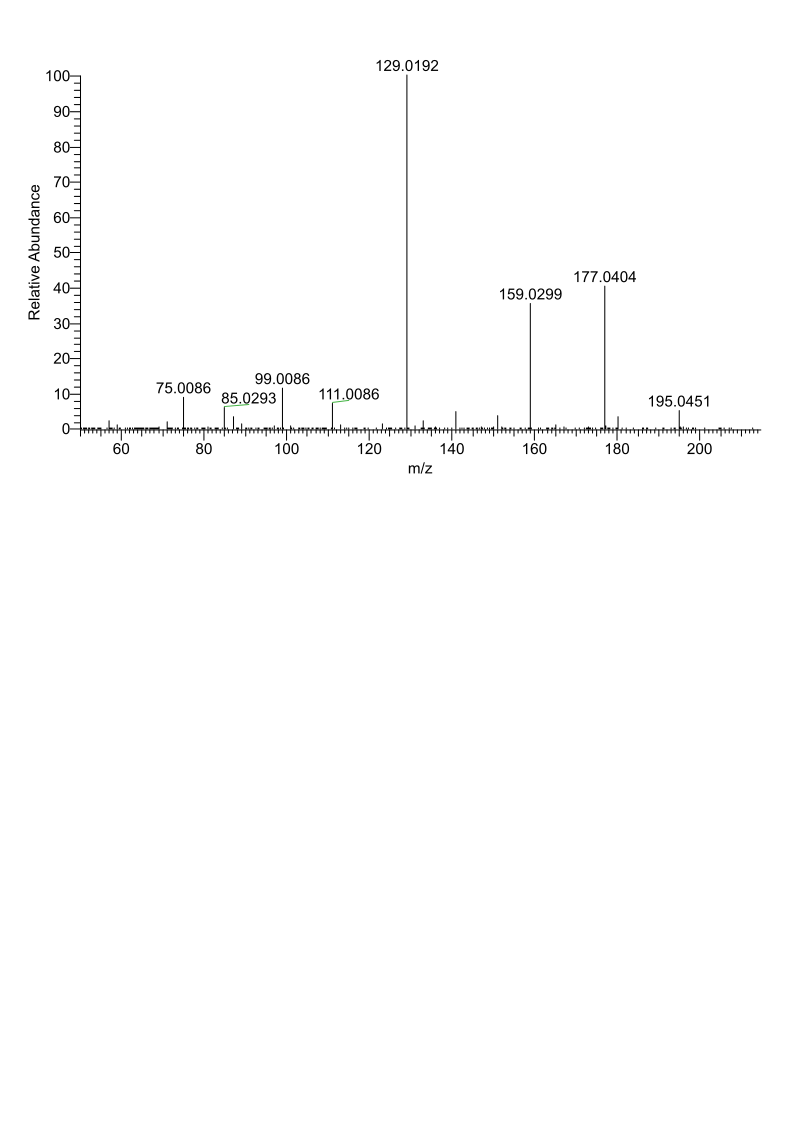


Figure S21b: Corresponding database MS^2^ spectrum for matched standard, D-gulonic acid.

**
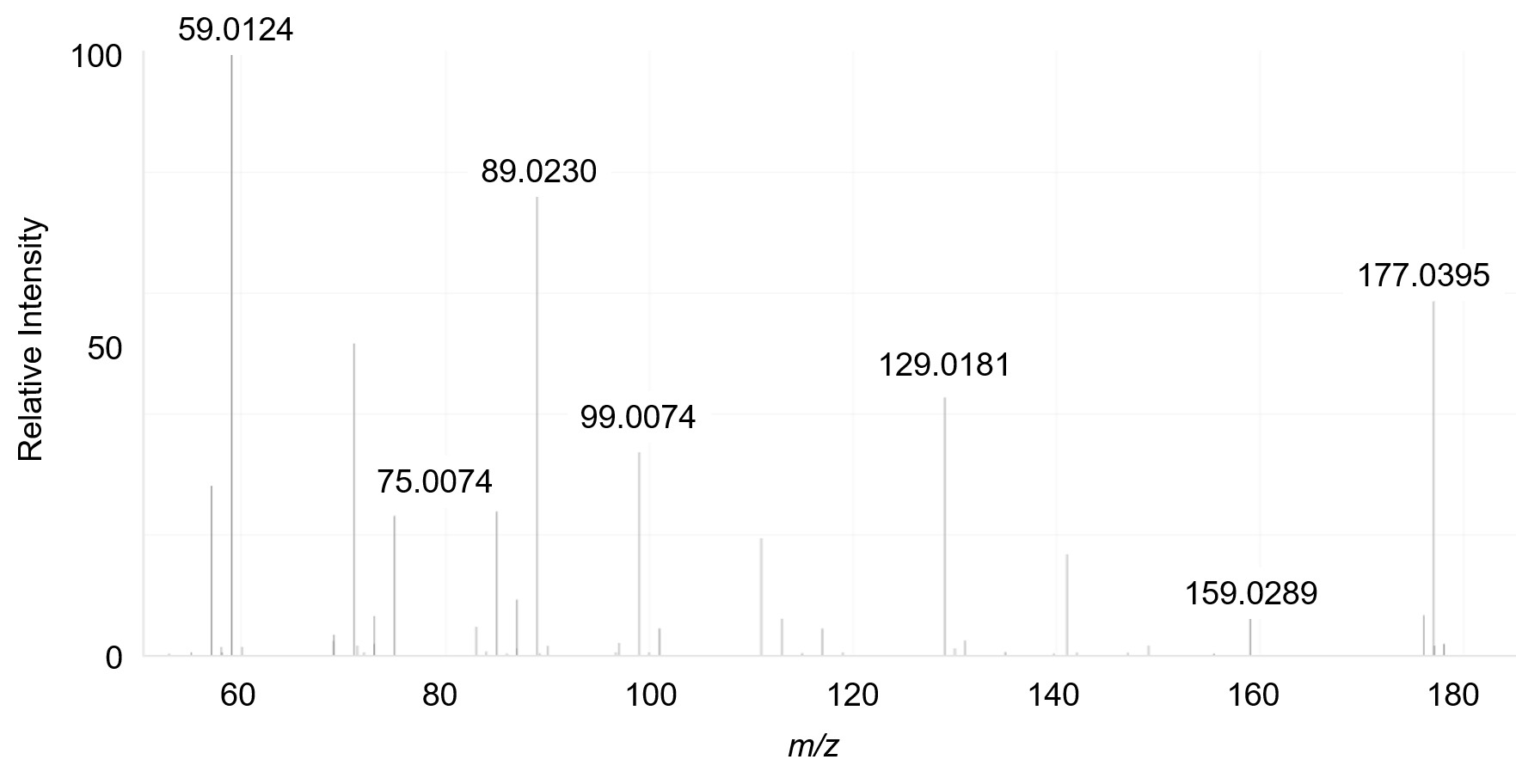
**

**Supplementary Tables**

Table S1: List of authentic standards (low molecular weight organic compounds) used to evaluate untargeted, high-resolution mass spectrometry technique.

| Compound | Class | Formula | Monoisotopic mass | Observed ion [M+H]^+^ | Mass accuracy (ppm) |
| --- | --- | --- | --- | --- | --- |
| Urea | Osmolyte | CH_4_N_2_O | 60.0318 | 61.0393 | 5.65 |
| Cytosine | Nucleobase (pyrimidine) | C_4_H_5_N_3_O | 111.0427 | 112.0497 | 7.55 |
| Betaine | Osmolyte | C_5_H_11_NO_2_ | 117.0784 | 118.0856 | 5.59 |
| Adenine | Nucleobase (purine) | C_5_H_5_N_5_ | 135.0539 | 136.0610 | 5.72 |
| Ectoine | Osmolyte | C_6_H_10­_N_2_O_2_ | 142.0737 | 143.0806 | 6.36 |
| Lysine | Amino acid (basic) | C_6_H_14_N_2_O_2_ | 146.1049 | 147.1120 | 5.50 |
| Glutamic Acid | Amino acid (acidic) | C_5_H_9_NO_4_ | 147.0526 | 148.0596 | 5.67 |
| Methionine | Amino acid (nucleophile) | C_5_H_11_NO_2_S | 149.0505 | 150.0575 | 5.54 |
| Arginine | Amino acid (basic) | C_6_H_14_N_4_O_2_ | 174.1111 | 175.1179 | 6.04 |
| N-acetyl glucosamine | Amino sugar | C_8_H_15_NO_6_ | 221.0894 | 222.0968 | 1.87 |
| Tetraglycine | Peptide | C_8_H_14_N_4_O_5_ | 246.0958 | 247.1019 | 7.29 |
| 6-methyl amino purine riboside | Nucleoside | C_11_H_15_N_5_O_4_ | 281.1118 | 282.1179 | 6.33 |
| Tyrosine-Phenylalanine | Dipeptide | C_18_H_20_N_2_O_4_ | 328.1418 | 329.1470 | 7.87 |
| YIGSR | Pentapeptide | C_26_H_42_N_8_O_8_ | 594.3126 | 595.3238 | 5.68 |
| Chlorophyll a^a^ | Pigment/ vitamin | C_55_H_72_MgN_4_O_5_ | 892.5353 | 871.5478 | 5.24 |

^a^Observed ion: [M-Mg+H]^+^

Table S2: List of abundant high-quality features that consistently (n = 2/3) and significantly (log_2_ fold change > 1.5, p-value < 0.05) varied between the top and bottom of the soil core and matched to a database within +/- 5 ppm. The LC/MS condition, detected [M+H]^+^ or [M-H]^-^ ion, CV% for peak areas across triplicate extracts at both depths, Δppm from the matched compound, the predicted formula, top hit from database, which database it was detected in, and the compound class are reported. The list is sorted first by LC/MS condition, and then in order of increasing *m/z*.

| **LC/MS Condition** | **Detected *m/z*** | **Δppm** | **Predicted Formula** | **Top Database Hit** | **Database** | **Class (description)** | **CV%** |
| --- | --- | --- | --- | --- | --- | --- | --- |
| HILIC (+) | 72.0807 | 1.07 | C_4_H_9_N | Pyrrolidine | HMDB | cyclic secondary amine; saturated heterocycle | 3.30 |
| HILIC (+) | 84.0807 | 0.92 | C_5_H_9_N | (+)-2,3-Dihydro-3- methyl-1H-pyrrole | HMDB | secondary amine; unsaturated aliphatic ring | 4.08 |
| HILIC (+) | 86.0963 | 1.52 | C_5_H_11_N | Piperidine | HMDB | heterocyclic amine | 2.12 |
| HILIC (+) | 87.044 | 0.71 | C_4_H_6_O_2_ | 2-Butenoate;2-Butenoic acid | MMCD | carboxylic acid | 3.14 |
| HILIC (+) | 90.0548 | 1.71 | C_3_H_7_NO_2_ | Alanine;2-Aminopropionic acid | MMCD | amino acid | 1.51 |
| HILIC (+) | 104.0705 | 1.04 | C_4_H_9_NO_2_ | Beta-alanine-methyl-ester | MMCD | amino acid | 2.07 |
| HILIC (+) | 115.0753 | 0.67 | C_6_H_10_O_2_ | Gamma-hexenoic acid | LIPID MAPS | lipid | 3.07 |
| HILIC (+) | 116.0705 | 0.93 | C_5_H_9_NO_2_ | D-Proline;L-Proline* | HMDB | amino acid; osmolyte | 3.01 |
| HILIC (+) | 120.0807 | 0.64 | C_8_H_9_N | Indoline* | MMCD | aromatic heterocycle; unsaturated; bicyclic | 3.54 |
| HILIC (+) | 132.1018 | 0.81 | C_6_H_13_NO_2_ | Alloisoleucine* | MMCD | amino acid | 1.42 |
| HILIC (+) | 138.0548 | 1.22 | C_7_H_7_NO_2_ | Benzhydroxamic acid | MMCD | aromatic | 2.41 |
| HILIC (+) | 146.0599 | 0.95 | C_9_H_7_NO | Quinolin-4-ol | MMCD | monohydroxyquinoline; alcohol | 2.73 |
| HILIC (+) | 162.1123 | 1.04 | C_7_H_15_NO_3_ | N-methyl-4-hydroxy-leucine | MMCD | N-methyl amino acid | 2.34 |
| HILIC (+) | 165.0697 | 1.11 | C_13_H_8_ | (E)-1,11-Tridecadiene-3,5,7,9-tetrayne | MMCD | hydroxy fatty acid, lipid | 2.10 |
| **LC/MS Condition** | **Detected *m/z*** | **Δppm** | **Predicted Formula** | **Top Database Hit** | **Database** | **Class (description)** | **CV%** |
| HILIC (+) | 166.0861 | 0.92 | C_9_H_11_NO_2_ | 4-(3-Pyridyl)-butanoic acid | MMCD | aromatic carboxylic acid | 2.44 |
| HILIC (+) | 167.9817 | 0.73 | C_3_H_4_O_6_P | Phosphoenol pyruvate; Phosphoenolpyruvic acid; PEP | MMCD | metabolite; ester, carboxylic acid | 3.37 |
| HILIC (+) | 176.1028 | 0.96 | C_6_H_13_N_3_O_3_ | Citrulline* | HMDB | carboxylic acid, imine | 2.57 |
| HILIC (+) | 182.0811 | 0.42 | C_9_H_11_NO_3_ | Beta-Tyrosine* | HMDB | amino acid | 4.21 |
| HILIC (+) | 184.0636 | 1.17 | C_5_H_13_NO_4_S | Choline sulfate | MMCD | quaternary amine, sulfate | 2.58 |
| HILIC (+) | 188.0705 | 0.70 | C_11_H_9_NO_2_ | N-(2,5-Dihydroxyphenyl) pyridinium* | HMDB | plant nutrient | 6.07 |
| HILIC (+) | 189.1232 | 0.89 | C_8_H_16_N_2_O_3_ | Glycyl-Isoleucine | HMDB | dipeptide | 3.76 |
| HILIC (+) | 204.0865 | 0.75 | C_8_H_13_NO_5_ | N2-acetyl-alpha-aminoadipate | MMCD | dicarboxylic acid, amide | 3.35 |
| HILIC (+) | 220.1178 | 0.70 | C_9_H_17_NO_5_ | Pantothenate; Pantothenic acid;(R)-Pantothenate* | MMCD | secondary alcohol | 2.13 |
| HILIC (+) | 226.9514 | 2.43 | C_6_H_4_Cl_2_O_5_ | 2,4-Dichloro-3-oxoadipate | MMCD | dicarboxylic acid, ketone, dihalide | 1.06 |
| HILIC (+) | 229.1545 | 0.80 | C_11_H_20_N_2_O_3_ | Leucyl-Proline | HMDB | dipeptide | 2.51 |
| HILIC (+) | 238.092 | 0.58 | C_8_H_15_NO_7_ | Fructoseglycine | MMCD | sugar, amino acid | 6.15 |
| HILIC (+) | 251.076 | 0.55 | C_9_H_14_O_8_ | 4,6-O-(1-carboxyethylidene)-beta-D-glucose | MMCD | sugar | 3.85 |
| HILIC (+) | 251.0761 | 0.18 | C_9_H_14_O_8_ | (4AR,6R,7S,8R,8AS)-hexahydro-6,7,8-trihydroxy-2-methylpyrano[3,2-D][1,3]dioxine-2-carboxylic acid | MMCD | lipid | 3.58 |
| HILIC (+) | 261.144 | 1.75 | C_11_H_20_N_2_O_5_ | (E)-N-6-[3-carboxy-1-(hydroxy methyl) propylidene]-L-lysine | MMCD | peptide | 6.98 |
| HILIC (+) | 265.1434 | 0.12 | C_15_H_20_O_4_ | 4-Hydroxy dehydromyo-porone | HMDB | aromatic, monoterpenoid | 2.56 |
| HILIC (+) | 304.1014 | 4.23 | C_12_H_17_NO_8_ | Gynocardin | HMDB | phytochemical | 2.74 |
| **LC/MS Condition** | **Detected *m/z*** | **Δppm** | **Predicted Formula** | **Top Database Hit** | **Database** | **Class (description)** | **CV%** |
| HILIC (+) | 365.1564 | 2.60 | C_14_H_24_N_2_O_9_ | N-Acetylmuramoyl-Ala;N-Acetyl-D-muramoyl-L-alanine | MMCD | amino acid | 2.77 |
| HILIC (+) | 453.2091 | 1.75 | C_24_H_30_F_2_O_6_ | 8-isobutanoyl-neosolaniol | MMCD | sesquiterpene; mycotoxin | 2.45 |
| HILIC (+) | 591.3864 | 4.65 | C_34_H_54_O_8_ | Lasalocid A | LIPID MAPS | lipid | 3.92 |
| HILIC (+) | 635.4124 | 4.62 | C_36_H_58_O_9_ | Maslinic acid | HMDB | triterpene saponin | 2.30 |
| HILIC (-) | 207.0333 | 0.15 | C_7_H_12_O_5_S | 3-(2'-methylthio) ethylmalic-acid | KEGG | plant metabolite; natural pesticide | 3.62 |
| HILIC (-) | 219.1021 | 2.56 | C_13_H_16_O_3_ | Ethyl 2-benzylacetoacetate* | HMDB | beta-ketoacid, plant metabolite | 4.12 |
| HILIC (-) | 227.1074 | 1.54 | C_10_H_16_N_2_O_2_ | Pyroglutamyl-valine* | HMDB | acidic dipeptide | 1.74 |
| HILIC (-) | 229.1239 | 2.19 | C_15_H_18_O_2_ | 8,12-Epoxy-4(15),7,11-eudesmatrien-1-one* | HMDB | metabolite; sesqui-terpenoid | 3.83 |
| HILIC (-) | 241.1231 | 1.26 | C_16_H_18_O_2_ | 4,4'-(Butane-1,1-diyl)diphenol;1,1-Bis(4-hydroxy phenyl)butane | MMCD | metabolite | 2.56 |
| HILIC (-) | 263.0968 | 1.62 | C_12_H_16_N_4_OS | 2,6-diamino-8-propylsulfanylmethyl-3H-quinazoline-4-one | MMCD | microbial metabolite | 4.17 |
| HILIC (-) | 265.0759 | 5.39 | C_16_H_12_NO_3_ | Ungeremine | MMCD | plant metabolite; betaine alkaloid; osmolyte; bactericide | 6.49 |
| HILIC (-) | 271.1231 | 3.59 | C_19_H_16_N_2_ | Sempervirine | MMCD | aromatic, amine | 3.46 |
| HILIC (-) | 287.0947 | 7.63 | C_16_H_16_O_5_ | Alkannin | MMCD | plant metabolite | 1.65 |
| HILIC (-) | 293.1442 | 4.67 | C_13_H_26_O_5_S | Heptyl 1-thiohexopyranoside* | MMCD | sugar; heteroatom | 1.36 |
| HILIC (-) | 311.0816 | 3.23 | C_14_H_26_Cl_2_O_2_ | Methyl dichlorotridecanoate | LIPID MAPS | lipid; fatty acid | 4.54 |
| HILIC (-) | 351.1502 | 7.02 | C_21_H_22_NO_4_ | Palmatine;5,6-Dihydro-2,3,9,10-tetramethoxydibenzo [a,g]quinolizinium | MMCD | metabolite | 4.44 |
| **LC/MS Condition** | **Detected *m/z*** | **Δppm** | **Predicted Formula** | **Top Database Hit** | **Database** | **Class (description)** | **CV%** |
| HILIC (-) | 371.1039 | 5.25 | C_19_H_20_N_2_O_4_S | 2-(1,3-dioxo-1,3-dihydro-2H-isoindol-2-YL) ethyl-4-(4'-ethoxy [1,1'-biphenyl-4-YL)-4-oxbutanoic acid | MMCD | metabolite | 3.77 |
| HILIC (-) | 457.1309 | 9.33 | C_20_H_26_O_12_ | 3-O-a-L-Arabinofuranosyl-D-xylose, 9CI; b-Pyranose-form, 2-O-(4-Hydroxy-3-methoxycinnamoyl)* | HMDB | plant sugar | 3.57 |
| RP (+) | 60.0444 | 0.19 | C_2_H_5_NO | Aminoacetaldehyde* | MMCD | alkylamine | 0.76 |
| RP (+) | 85.0284 | 0.09 | C_4_H_4_O_2_ | 4-Hydroxy-2-butenoic acid gamma-lactone | HMDB | organic acid | 2.00 |
| RP (+) | 101.0709 | 0.35 | C_4_H_8_N_2_O | N-nitroso-pyrrolidine | HMDB | basic heterocycle; secondary amine | 1.27 |
| RP (+) | 148.0602 | 1.66 | C_5_H_9_NO_4_ | Glutamic acid | KEGG | metabolite | 2.41 |
| RP (+) | 176.103 | 0.17 | C_6_H_13_N_3_O_3_ | D-Citrulline* | MMCD | amino acid | 4.18 |
| RP (+) | 212.1644 | 0.50 | C_12_H_21_NO_2_ | Elaeokanine C | PubChem | alkaloid | 2.95 |
| RP (+) | 216.1958 | 0.07 | C_12_H_25_NO_2_ | 12-amino-dodecanoic acid | LIPID MAPS | carboxylic acid, amine | 3.30 |
| RP (+) | 226.1285 | 0.06 | C_8_H_19_NO_6_ | 5-deoxy-5-[(1S)-1-hydroxyethyl]amino-D-glucitol | MMCD | sugar | 3.33 |
| RP (-) | 195.0512 | 0.86 | C_6_H_12_O_7_ | L-Gulonate;L-Gulonic acid;Gulonate* | MMCD | sugar-derivative | 11.18 |
| RP (-) | 269.2493 | 2.48 | C_17_H_34_O_2_ | 15-methyl palmitic acid | LIPID MAPS | lipid | 4.93 |

*Indicates experimental and database MS^2^ information shown in figures above (S7-S21b).

Table S3: Mobile phase conditions and additives that were tested to optimize each LC phase and MS polarity. Final mobile phase compositions are shown in bold.

| **HILIC (+)** | | |
| --- | --- | --- |
| **A: 60 % ACN, 40 % NH_4_Ac, 0.1 % FA** | **B: 95 % ACN, 5 % NH_4_Ac, 0.1 % FA** | pH |
| 2.5 mM NH_4_Ac | 2.5 mM NH_4_Ac | 3.2 |
| **5 mM NH_4_Ac** | **5 mM NH_4_Ac** | **3.5** |
| 10 mM NH_4_Ac | 10 mM NH_4_Ac | 3.7 |
| 20 mM NH_4_Ac | 20 mM NH_4_Ac | 4.0 |
| **HILIC (-)** | | |
| **A: 100 % NH_4_Ac, 0.1 % NH_4_OH** | **B: 95 % ACN, 5 % NH_4_Ac, 0.1 % NH_4_OH** | pH |
| 2.5 mM NH_4_Ac |  | 9.0 |
| **5 mM NH_4_Ac** | **5 mM NH_4_Ac** | **9.1** |
| 10 mM NH_4_Ac |  | 9.2 |
| 20 mM NH_4_Ac |  | 9.3 |
| **RP (+)** | | |
| **A: 95 % H_2_O, 5 % ACN** | **B: 70 % ACN, 30 % H_2_O** | pH |
| **0.1 % FA** | **0.1 % FA** | **3.5** |
|  |  |  |
| **RP (-)** | | |
| A: 97 % H_2_O, 3 % MeOH | B: 100 % MeOH | pH |
| 20 µM TEAB* | 20 µM TEAB* | 8.3 |
| 15 mM acetic acid |  | 5.0 |
|  |  |  |
| **A: 90 % H_2_O, 10 % IPA** | **B: 80 % ACN, 10 % H_2_O, 10 % IPA** | pH |
| **1 mM NH_4_OH** | **1 mM NH_4_OH** | **9.0** |

*tetraethylammonium bromide

Table S4: Optimized gradient conditions for nano-LC separations, for positive- and negative-MS-ionization modes on C18-RP and ZIC-pHILIC columns.

| C18 Reversed-Phase | | | | |  | ZIC-pHILIC | | | | |
| --- | --- | --- | --- | --- | --- | --- | --- | --- | --- | --- |
| Positive | |  | Negative | |  | Positive | |  | Negative | |
| time, min | % B |  | time, min | % B |  | time, min | % A |  | time, min | % A |
| 0.0 | 2 |  | 0.0 | 25 |  | 0.0 | 0 |  | 0.0 | 0 |
| 3.0 | 2 |  | 3.0 | 25 |  | 3.0 | 0 |  | 3.0 | 0 |
| 23.0 | 100 |  | 23.0 | 100 |  | 23.0 | 100 |  | 23.0 | 30 |
| 28.0 | 100 |  | 28.0 | 100 |  | 28.0 | 100 |  | 28.0 | 30 |
| 33.0 | 2 |  | 33.0 | 25 |  | 30.0 | 80 |  | 30.0 | 60 |
| 40.0 | 2 |  | 40.0 | 25 |  | 35.0 | 80 |  | 35.0 | 60 |
|  |  |  |  |  |  | 40.0 | 0 |  | 40.0 | 0 |
|  |  |  |  |  |  | 45.0 | 0 |  | 45.0 | 0 |

**Supplementary References**

1. Hu, M.; Krauss, M.; Brack, W.; Schulze, T., Optimization of LC-Orbitrap-HRMS acquisition and MZmine 2 data processing for nontarget screening of environmental samples using design of experiments. *Anal. Bioanal. Chem.* **2016,** *408* (28), 7905-7915.

2. Nordstrom, A.; O'Maille, G.; Qin, C.; Siuzdak, G., Nonlinear data alignment for UPLC-MS and HPLC-MS based metabolomics: Quantitative analysis of endogenous and exogenous metabolites in human serum. *Anal. Chem.* **2006,** *78* (10), 3289-3295.

3. Pluskal, T.; Castillo, S.; Villar-Briones, A.; Oresic, M., MZmine 2: Modular framework for processing, visualizing, and analyzing mass spectrometry-based molecular profile data. *BMC Bioinformatics* **2010,** *11*.

4. Polpitiya, A. D. Q., W.J.; Jaitly, N.; Petyuk, V.A.; Adkins, J.N.; Camp, D.G. 2nd; Anderson, G. A. S., R.D., DanTE: a statistical tool for quantitative analysis of -omics data. *Bioinformatics* **2008,** *24* (13), 1556-1558.

5. Tyanova, S.; Temu, T.; Sinitcyn, P.; Carlson, A.; Hein, M. Y.; Geiger, T.; Mann, M.; Cox, J., The Perseus computational platform for comprehensive analysis of (prote)omics data. *Nat. Methods* **2016,** *13* (9), 731-740.
